# The efficacy of gut microbiota-regulating drugs on metabolic dysfunction-associated steatotic liver disease: a systematic review and network meta-analysis

**DOI:** 10.7717/peerj.21166

**Published:** 2026-04-20

**Authors:** Yi Wang, Jiqi Ouyang, Houyan Zhang, Yaocun Shen, Wenliang Lv

**Affiliations:** 1 Graduate School of Beijing University of Chinese Medicine, Beijing, China; 2 Department of Infection, Guang’an Men Hospital, China Academy of Chinese Medical Sciences, Beijing, China; 3 Shandong First Medical University, Jinan, Shandong, China

**Keywords:** Metabolic dysfunction-associated steatotic liver disease, Randomized controlled trial, Gut microbiota, Meta-analysis

## Abstract

**Objective:**

Currently, effective drugs for metabolic dysfunction-associated steatotic liver disease (MASLD) are limited, with treatment primarily focusing on diet and exercise. Recent studies suggest that gut microbiota-regulating drugs may offer therapeutic benefits. Therefore, our aim is to evaluate the efficacy of these medications in MASLD patients.

**Methods:**

This systematic review and network meta-analysis involved a search of PubMed, Web of Science, Embase, Cochrane Library, and ClinicalTrials.gov for randomized controlled trials (RCTs) published from January 1, 2012, to January 28, 2026. The intervention measures encompassed probiotics, prebiotics, synbiotics, antibiotics, postbiotics, and a combination of antibiotics and gut microbiota-regulating drugs. The control group received a placebo or usual care. The risk of bias in the included research was evaluated utilizing the revised Cochrane risk of bias tool for randomised trials. The confidence of evidence will be evaluated through the CINeMA (Confidence in Network Meta-Analysis) web application. Liver enzymes and hepatic steatosis were taken as the primary outcome and were analyzed by random-effects, Bayesian network meta-analyses. A two stage network meta-analysis harnessing the surface under the cumulative ranking curve (SUCRA) was performed for assessing the comparative efficacy of medications classes and particular gut microbiota-regulating drugs. The study was registered on PROSPERO: CRD42024606333.

**Results:**

A total of 27 studies comprising 1,511 participants were included in this meta-analysis. Relative to placebo, reductions in alanine aminotransferase (ALT) levels were observed with probiotics (Mean Difference (MD): −7.51, 95% credible intervals (CI) [−12.36 to −2.66]), prebiotics (MD: −13.64, 95% CI [−27.07 to −0.22]) and antibiotics (MD: −24.30, 95% CI [−47.02 to −1.58]). Probiotics (MD: −6.42, 95% CI [−11.91 to −0.92]) and synbiotics (MD: −13.13, 95% CI [−20.82 to −5.45]) were both associated with a reduction in aspartate aminotransferase (AST) levels compared to placebo. Declines in gamma-glutamyl transferase (GGT) levels (MD: −12.40, 95% CI [−23.13 to −1.68]) were observed with synbiotics compared to the placebo. Additionally, synbiotics significantly reduced controlled attenuation parameter (CAP) levels compared with placebo (MD: −45.69, 95% CI [−56.39 to −34.99]). However, no statistically significant differences were observed between probiotics and synbiotics with respect to lipid markers total cholesterol (TC), triglycerides (TG), high-density lipoprotein cholesterol (HDL-C), or inflammatory cytokines tumor necrosis factor alpha (TNF-α) and interleukin 6 (IL-6).

**Conclusions:**

Probiotics and synbiotics significantly improve liver enzymes and hepatic steatosis in patients with MASLD.

## Introduction

Metabolic dysfunction-associated steatotic liver disease (MASLD), formerly known as non-alcoholic fatty liver disease (NAFLD), has become the predominant cause of chronic liver disease in the developed world, with its prevalence rapidly increasing (Estes et al., 2018). MASLD plays a major role in the global burden of liver diseases and is projected to become the leading cause of end-stage liver disease in the coming decades. The epidemiology and demographic characteristics of MASLD vary across regions, often correlating with the prevalence of obesity (Younossi et al., 2018). MASLD encompasses both simple steatosis and metabolic dysfunction-associated steatohepatitis (MASH), the latter of which can progress to liver fibrosis, cirrhosis, and hepatocellular carcinoma (Feng et al., 2024; Hsu & Loomba, 2024). Current pharmacological treatments show limited effectiveness, and the disease burden remains substantial, the critical need for effective therapeutic targets (Xue et al., 2022). Recent studies have increasingly concentrated on the interaction between the gut and liver. The liver, an essential organ in digestion, is continuously exposed to nourishment, toxic substances, and compounds from the gut microbiota *via* the bloodstream (Ji et al., 2019). Dysregulation of the gut microbiota is a key feature of MASLD, with specific microbial signatures correlating with disease severity through altered bacterial metabolites (Chen & Vitetta, 2020). Several mechanisms have been proposed linking the gut microbiome to MASLD, involving dysbiosis of the intestinal endothelium barrier, which results in the translocation of bacterial components and subsequently causes liver inflammation (Safari & Gérard, 2019). Furthermore, metabolites generated by the intestinal microbiota can influence liver function. Indigestible carbohydrates, such as dietary fiber, are fermentable by gut microbiota, leading to the production of important metabolites, particularly short-chain fatty acids and succinate. Evidence from animal models and certain human clinical trials suggests that these substances contribute to reducing the incidence and managing MASLD (Canfora et al., 2019). Extensive research has focused on gut microbiota modulation as a therapeutic strategy for this condition.

Medication approaches to improving MASLD by gut microbiota manipulation comprise multiple options, including probiotics, prebiotics, antibiotics, and others. Numerous promising animal studies confirm that gut microbiota-regulating administration can produce advantageous impacts on MASLD progression (Ye et al., 2018; Chappuis et al., 2017; Janssen et al., 2017). Based on current clinical studies, several meta-analyses have been conducted to figure out the influence of interventions targeting gut microbiota regulation on MASLD (Rong et al., 2023; Khan et al., 2019). Recent review studies (Ntikoudi et al., 2025) have also suggested that probiotics, prebiotics, and synbiotics represent promising approaches for modulating the intestinal flora, with the potential to exert beneficial effects in patients with MASLD. The results indicate that treatments aimed at regulating the gut microbiota are linked to substantial enhancements in liver-related outcomes for those with MASLD. Although these studies include various methods of regulating gut microbiota, they use the traditional two-group comparison approach and do not compare the effectiveness of different methods. Network meta-analysis is a statistical method grounded in Bayesian or classical statistical theory, developed as an extension of traditional meta-analysis. Constructing a network structure allows for the integration of both direct and indirect comparison data, facilitating a comprehensive evaluation, ranking, and comparison of the efficiency and safety in various medical treatments. Traditional meta-analyses are typically confined to direct pairwise comparisons, allowing for a summary of outcomes related to a single intervention. However, such an approach is limited because it cannot assess multiple therapeutic strategies simultaneously. Network meta-analysis can assess three or more interventions. Through the establishment of a network framework, it enables comprehensive comparisons and rankings across several interventions, thereby providing a more robust evidence base for evaluating efficacy and safety. Although there have been network meta-analyses investigating the use of synbiotics, probiotics, and prebiotics for the treatment of MASLD (Kanchanasurakit et al., 2022), similar to traditional meta-analyses, their primary outcome has focused on improvements in enzymatic levels, without considering the role of postbiotics or assessing the imaging parameter of controlled attenuation parameter (CAP). As MASLD progresses, hepatic fat accumulation increases in parallel with disease severity. Consequently, the precise quantification of hepatic steatosis serves as a crucial metric for assessing both disease severity and therapeutic efficacy. Among various measurement techniques, CAP is a non-invasive imaging modality for quantifying liver fat and is widely utilized in evaluating disease prognosis. Hence, we utilize network meta-analysis to systematically compare the efficacy of different gut microbiota-regulating drugs, with the objective of evaluating and contrasting the effectiveness of these approaches. This study provides an evidence-based foundation for the clinical selection of gut microbiota-regulating drugs. Specifically, we explored the alterations in hepatic steatosis, liver enzymes, and inflammatory factors.

## Materials & Methods

This systematic review and network meta-analysis was conducted and reported in accordance with the Preferred Reporting Items for Systematic Reviews and Meta-analyses (PRISMA) guidelines to ensure the dependability and integrity of the content and conclusions. The trial registration number CRD42024606333 was assigned to this study on the International Prospective Register of Systematic Reviews (PROSPERO).

### Data sources and search methodology

The search for literature was performed using English databases, in particular PubMed, Web of Science, Embase, and Cochrane Library, and ClinicalTrials.gov covering the period from January 1, 2012, to January 28, 2026. Two reviewers (Yi Wang and Jiqi Ouyang) independently performed a literature search and evaluated the titles and abstracts of the identified studies based on the established inclusion criteria. When abstracts provided insufficient information, the full texts were reviewed to support decision-making. The third reviewer (Yaocun Shen) assessed the disagreements between the two reviewers.

Probiotics were searched with ‘probiotics’; prebiotics was searched with ‘prebiotics’; synbiotics were searched with ‘synbiotics’; postbiotics were searched with ‘postbiotics’; antibiotics were searched with ‘antibiotics’. MASLD was searched with ‘non-alcoholic fatty liver disease’, ‘metabolic associated fatty liver disease’, and ‘metabolic dysfunction-associated steatotic liver disease’. Gut microbiota-regulating drugs and MASLD were utilized with the logical operator “AND”. The specific search strategy used was: (probiotics OR prebiotics OR synbiotics OR postbiotics OR antibiotics) AND (“non-alcoholic fatty liver disease” OR “NAFLD” OR “metabolic associated fatty liver disease” OR “MAFLD” OR “metabolic dysfunction-associated steatotic liver disease” OR “MASLD”). As search strategies vary across databases, the detailed search strings for each individual database are provided in Supplemental Information 2, p26. Eligible literature underwent secondary screening through title and abstract review to identify clinical studies of interest. Full-text assessment was subsequently performed to select research meeting predefined inclusion criteria.

### Study selection criteria

The studies included in this research involved patients with MASLD. MASLD is defined by hepatic steatosis (confirmed by liver biopsy, imaging, or serum biomarkers) combined with obesity, T2DM, or metabolic dysfunction. While serum biomarkers and imaging modalities are integral to clinical assessment, liver biopsy remains the gold standard for characterizing pathological subtypes. The interventions included probiotics, prebiotics, synbiotics, antibiotics, postbiotics, and antibiotics combined with gut microbiota-regulating drugs (combination treatment). In the subsequent data synthesis, antibiotics combined with gut microbiota-regulating drugs were analyzed as an independent intervention group. The control group received standard treatment. The outcomes assessed in this study included liver enzymes, such as alanine aminotransferase (ALT), aspartate aminotransferase (AST), and gamma-glutamyl transferase (GGT); liver steatosis measured by CAP; lipid profile markers, including total cholesterol (TC), triglycerides (TG), high-density lipoprotein cholesterol (HDL-C), and low-density lipoprotein cholesterol (LDL-C); and inflammatory cytokines, including tumor necrosis factor alpha (TNF-α) and interleukin 6 (IL-6). All included studies were randomized controlled trials (RCTs) published in English.

We excluded letters, case reports, and reviews. Studies using non-pharmacological interventions, such as fecal microbiota transplantation, were also not included. Studies in which the outcome measures could not be extracted due to lack of original data were excluded. Studies that did not explicitly exclude hepatic steatosis arising from hepatotoxic drugs, alcohol, or pregnancy were also excluded.

### Data extraction

The eligible literature was determined based on the inclusion criteria, after which the researchers (Yi Wang and Jiqi Ouyang) independently reviewed the complete text and extracted data with precision. Disagreements were addressed *via* discussion and consensus involving a third researcher (Yaocun Shen). For clinical characteristics, we extracted the author, publication year, study duration, diagnostic criteria, subject characteristics, intervention types, and specific strains, as well as the treatment dosage and frequency, and treatment duration. For continuous outcomes, we extracted the mean and standard deviation (SD) of the changes from baseline to the study endpoint for each group. In cases where the mean and SD were not provided, the estimated outcome was derived from the median, interquartile range, or range (Wu et al., 2024). A multivariate random-effects network meta-analysis (mvmeta in Stata) was performed to adjust for correlations among effect estimates in multi-arm studies. By correctly estimating the covariance structure, this model avoids participant double-counting. For any multi-arm studies included, interventions were categorized accordingly to ensure precise comparative analysis within the framework. Adverse events reported for all interventions will be systematically extracted and synthesized according to the specific treatment arms.

The risk of bias tool developed by the Cochrane Collaboration was employed to assess bias in the included RCTs. Two researchers (Yi Wang and Jiqi Ouyang) retrieved appropriate details and performed independent evaluations of bias risk. Discrepancies have been cleared up *via* clarification and validated by the additional researcher (Wenliang Lv). The quality of evidence for each outcome will be assessed using the CINeMA (Confidence in Network Meta-Analysis) web application. The CINeMA includes six domains: (1) within-study bias, (2) reporting bias, (3) indirectness, (4) imprecision, (5) heterogeneity, and (6) incoherence. Based on these domains, the confidence rating will be graded as high, moderate, low, or very low.

### Data synthesis and statistical analysis

In this study, liver enzymes (ALT, AST, and GGT) and hepatic steatosis (CAP) were defined as the primary outcomes, while lipid profiles (TC, TG, HDL-C, and LDL-C) and inflammatory markers (TNF-α and IL-6) were considered secondary outcomes. Continuous outcomes were expressed as mean differences (MD) with 95% confidence intervals (CI), and when different, non-standardized scales were used across studies, standardized mean differences (SMD) were calculated. Global, local, and loop inconsistency tests were applied to assess heterogeneity across studies, with *p* < 0.05 indicating statistically significant inconsistency. To compare and synthesize the relative efficacy of multiple interventions, a Bayesian network meta-analysis was performed using a hierarchical random-effects model based on Markov Chain Monte Carlo (MCMC) methods, thereby incorporating both direct and indirect evidence. Multiple parallel chains with adequate burn-in periods and iterations were specified, and convergence was confirmed when the Gelman–Rubin diagnostic value was <1.1. The relative efficacy of each intervention was ranked using ranking probabilities and surface under the cumulative ranking curve (SUCRA). The forest plots were applied to illustrate the effect size corresponding to each study and treatment. All analyses were conducted in Stata/MP version 18.0 using the network and bayesmh commands, with detailed codes provided alongside the raw data in the supplementary materials submitted with the manuscript.

## Results

### Study selection

**Figure 1:**
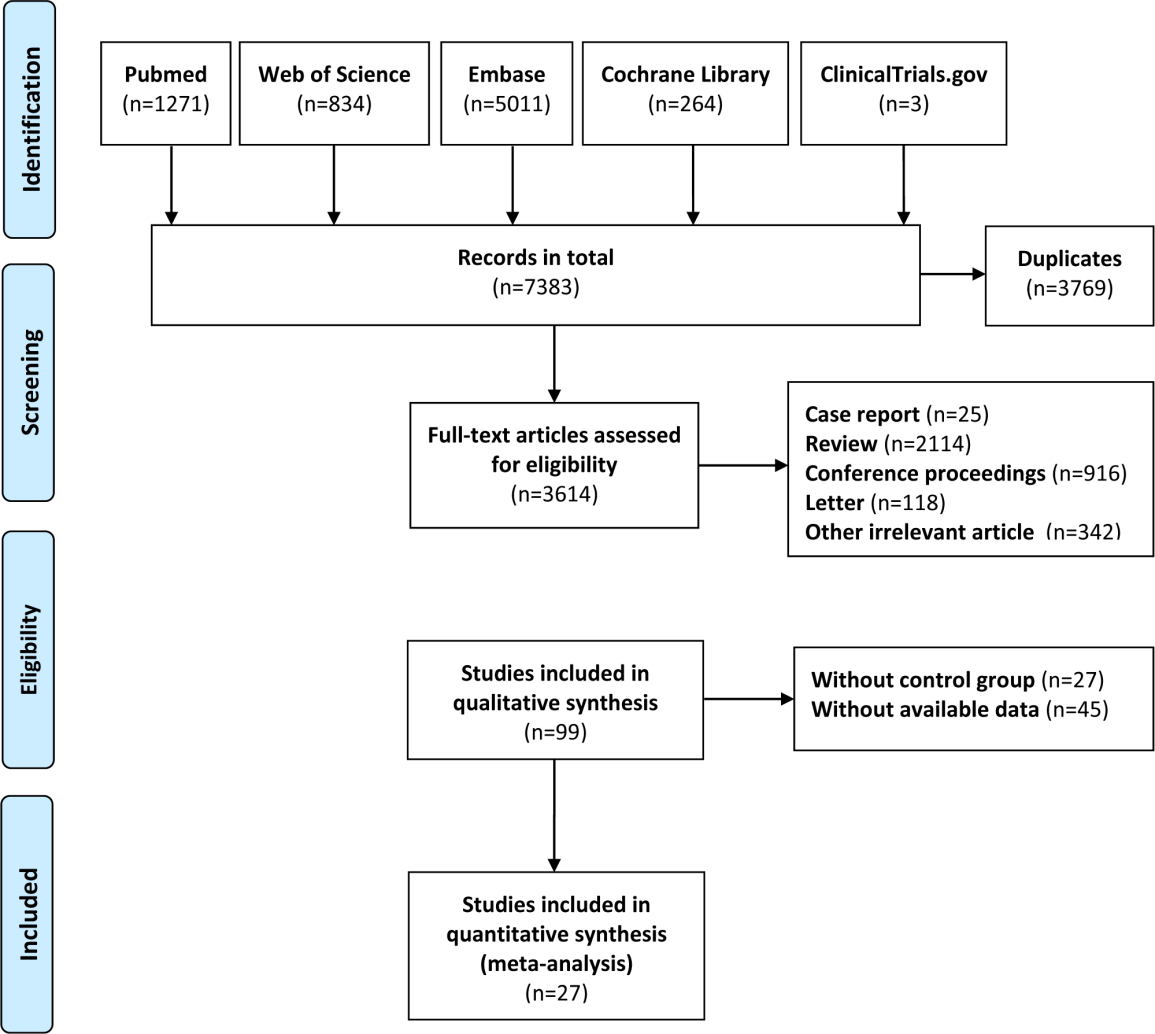
The literature screening process of the study. Introduction of index abbreviations: ALT, alanine aminotransferase; AST, aspartate aminotransferase; GGT, gamma-glutamyl transferase; CAP, controlled attenuation parameter; TC, total cholesterol; TG, triglycerides; HDL-C, high-density lipoprotein cholesterol; LDL-C, low-density lipoprotein cholesterol; TNF-α, tumor necrosis factor alpha; IL-6, interleukin 6.

**Figure 2:**
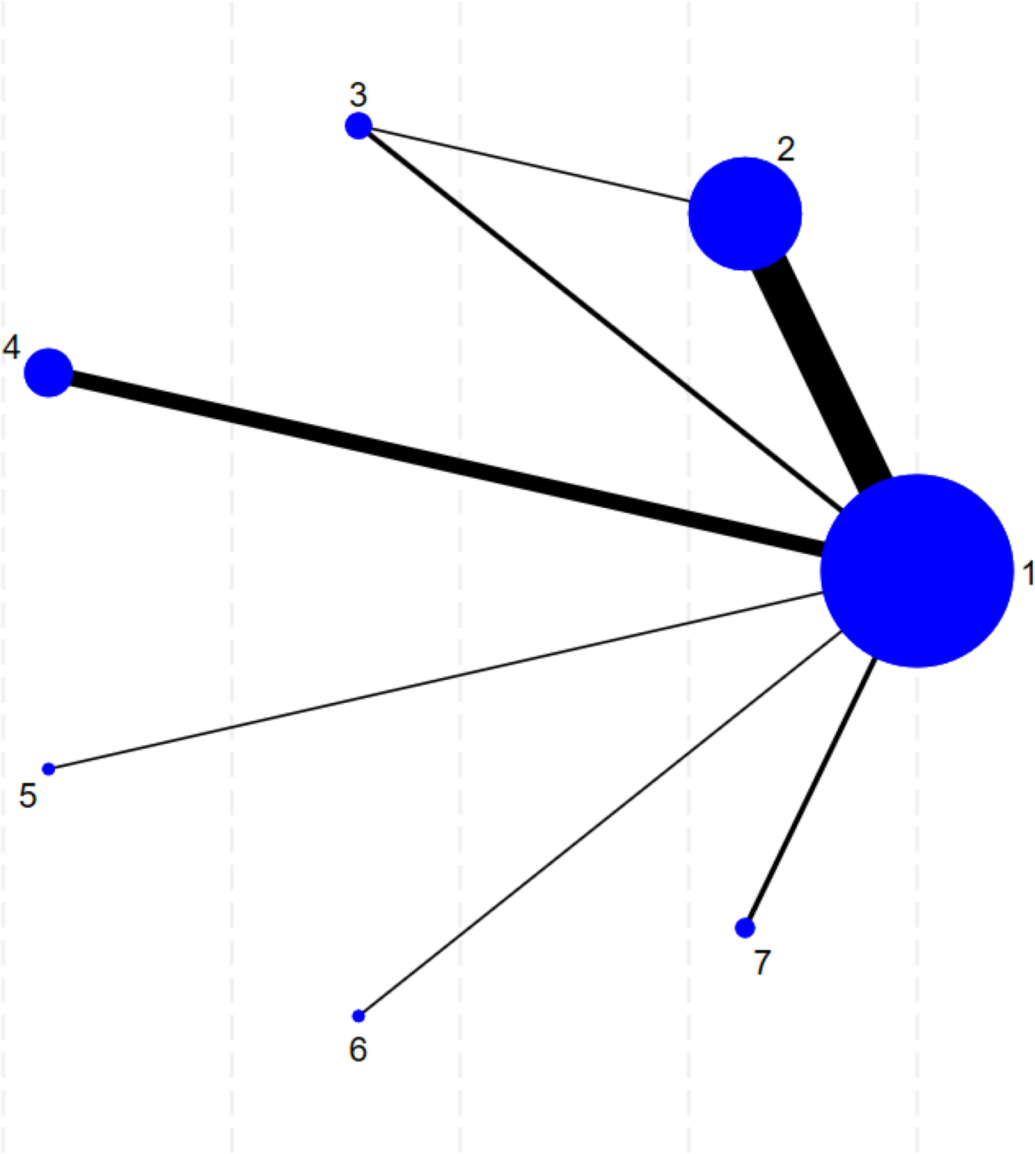
The network of the study. Each vertex in the network graph represents a distinct intervention. The size of the nodes corresponds to the total sample size for each intervention, while the thickness of the connecting edges indicates the number of studies comparing each pair of interventions. Intervention abbreviations: A, placebo or usual care controls; B, probiotics; C, prebiotics; D, synbiotics; E, antibiotics; F, postbiotics; G, combination of antibiotics and gut microbiota-regulating drugs (combination treatment).

After the full-text evaluation, a total of 27 RCTs evaluating the treatment of MASLD by gut microbiota-regulating drugs (Mohamad Nor et al., 2021; Didyk et al., 2024; Escouto et al., 2023; Famouri et al., 2017; Kobyliak et al., 2018; Chong et al., 2021; Crommen et al., 2022; Wong et al., 2013; Abhari et al., 2020; Alisi et al., 2014; Behrouz et al., 2020; Nabavi et al., 2014; Abd El Hamid et al., 2024; Sepideh et al., 2016; Manzhalii et al., 2017; Sayari, Neishaboori & Jameshorani, 2018; Eslamparast et al., 2014; Mofidi et al., 2017; Reshef et al., 2024; Chong et al., 2020; Ferolla et al., 2016; Abdel-Razik et al., 2018; Fogacci et al., 2024; Savytska et al., 2025; Won et al., 2025; Yazdani et al., 2025; Mohammadi et al., 2025). Figure 1 illustrates the literature screening process, following the review, evaluation, and discussion according to the inclusion and exclusion criteria. The electronic search manufactured 7,383 unique records. Screening and comprehensive analysis of full-text articles revealed 27 trials pertaining to 1,511 patients with interventions involving a total of six gut microbiota-regulating drugs. The evidence network for various outcome indicators is presented in Fig. 2. The sample size for the trial varied between 19 and 138 participants. A total of 15 studies evaluating the effects of probiotics and six studies on synbiotics were included. A study analyzed the effects of different gut microbiota-regulating drugs on MASLD at the same time (Behrouz et al., 2020). Probiotic or synbiotic interventions lasted between 8 and 28 weeks. Most of the studies used placebo or usual care as the control. A study evaluated the administration of antibiotics and probiotics as supplemental treatments with metformin, where both the intervention and control groups were treated with metformin (Didyk et al., 2024). Table 1 summarizes the fundamental characteristics of the included studies. To enhance transparency regarding potential effect modifiers relevant to transitivity, we summarized the key effect modifiers across the treatment arms. Baseline characteristics, including the presence of T2DM and the use of statins, metformin, and other concomitant medications, were generally comparable among the arms. A detailed comparison of these effect modifiers between treatment groups is presented in Supplemental Information 2 (‘Table S4’). Overall, most gut microbiota-regulating drugs demonstrated a favorable safety profile with low adverse event rates comparable to the control group, primarily consisting of mild gastrointestinal symptoms. The summary of adverse events for all interventions is presented in Supplemental Information 2 (‘Table S6’).

**Table 1:** The details of included RCTs.

**Author** **/year**	**Size**	**Duration of treatment (months)**	**Intervention group age**	**Control group age**	**Study type**	**Interventions (no.)**	**Strains** **(CFU dose)**	**Comparisons**	**Outcomes**	**Diagnosis in population**	**Methods of diagnosis**
Mohamad Nor et al., 2021 (NCT04074889)	39	6	54.70 ± 10.19	52.47 ± 16.73	RCT	Probiotics (6g/d) (*n* = 17)	Lactobacillus and Bifidobacterium (3.0 × 10^10^ CFU)	Probiotics *vs* placebo	ALT, AST, GGT, CAP, LSM, TC, TG	MASLD	Non-invasive imaging and serology
Didyk et al., 2024	68	5	59.8 ± 3.4	58.6 ± 2.9	RCT	Metformin and antibiotics combination (rifaximin, probiotics and prebiotics) (probiotics: 2 capsules /d; prebiotic: inulin and pectin, 12g/d) (*n* = 34)	Lactobacillus (7.0 × 10^9^ CFU), Bifidobacterium (1.0 × 10^9^ CFU), Saccharomyces (2.0 × 10^9^ CFU)	Metformin and antibiotics combination *vs* metformin	ALT, AST, IL-6	MASLD and T2DM	Non-invasive imaging and serology
Escouto et al., 2023 (NCT02764047)	48	6	58	57	RCT	Probiotics (1 capsule/d ) (*n* = 23)	Lactobacillus (1.0 × 10^9^ CFU) and Bifidobacterium (1.0 × 10^9^ CFU)	Probiotics *vs* placebo	ALT, AST, HDL-C, LDL-C, TC, TG, TNF-α	MASLD (MASH)	Liver biopsy
Famouri et al., 2017 (IRCT2013100414882N1)	64	3	12.7 ± 2.2	12.6 ± 1.7	RCT	Probiotics (1 capsule/d) (*n* = 32)	Lactobacillus (5 ×10^9^CFU) and Bifidobacterium (8 ×10^9^ CFU)	Probiotics *vs* placebo	ALT, AST, HDL-C, LDL-C, TC, TG	MASLD	Non-invasive imaging
Kobyliak et al., 2018 (NCT03434860)	58	2	53.4 ± 9.55	57.29 ± 10.45	RCT	Probiotics (10g/d) (*n* = 30)	Lactobacillus + Lactococcus (6 ×10^10^ CFU/g), Bifidobacterium (1 ×10^10^/g), Propionibacterium (3 ×10^10^/g), Acetobacter (1 ×10^6^/g) genera.	Probiotics *vs* placebo	ALT, AST, GGT, HDL-C, LDL-C, TC, TG, IL-6	MASLD	Non-invasive imaging and serology
Chong et al., 2021 (ISRCTN05474560)	35	2.5	57 ± 8	58 ± 7	RCT	Probiotics (4 sachets /d) (*n* = 19)	Lactobacillus, Bifidobacterium, and Streptococcus (4.5 ×10^11^ CFU)	Probiotics *vs* placebo	ALT, AST, HDL-C, LDL-C, TC, TG	MASLD	Liver biopsy and non-invasive imaging
Crommen et al., 2022 (NCT03585413)	48	3	40 ± 11	41 ± 9	RCT	Probiotics (3 capsules/d) and micronutrient (*n* = 25)	Lactobacillus, Bifidobacterium, Lactococcus, and Streptococcus (15 × 10^9^ CFU/4 g)	Probiotics and micronutrient *vs* placebo and micronutrient	ALT, AST, GGT, HDL-C, LDL-C, TC, TG, IL-6	MASLD	Serology
Wong et al., 2013 (NCT00870012)	20	6	42 ± 9	55 ± 9	RCT	Probiotics (20g/d) (*n* = 10)	Lactobacillus and Bifidobacterium (2 ×10^8^ CFU)	Probiotics *vs* placebo	ALT, AST, HDL-C, LDL-C, TC, TG, LSM	MASLD (MASH)	liver biopsy
Abhari et al., 2020 (IRCT20100524004010N23)	45	3	47.7 ± 11.4	46.7 ± 12.4	RCT	Synbiotics (1 capsule /d and inulin 0.4g/d) (*n* = 22)	Bacillus (1 × 10^9^ CFU)	Synbiotics *vs* placebo	ALT, AST, GGT, CAP, LSM, TNF-α, HDL-C, LDL-C, TC, TG	MASLD	Non-invasive imaging and serology
Alisi et al., 2014 (NCT01650025)	44	4	10 ± 2.22	11 ± 1.48	RCT	Probiotics (1 sachet/d) (*n* = 22)	Lactobacillus, Bifidobacterium, and Streptococcus (4.5 ×10^11^ CFU)	Probiotics *vs* placebo	ALT, TG	MASLD	Liver biopsy, non-invasive imaging and serology
Behrouz et al., 2020 (IRCT201410052394N13)	89	3	probiotic 38.46 ± 7.11/ prebiotic 38.41 ± 9.21	38.43 ± 10.09	RCT	Probiotics (1 capsule/d)(*n* = 30), prebiotics (16g/d) (*n* = 29)	Probiotics: Lactobacillus and Bifidobacterium) (5 × 10^9^ CFU); prebiotics: Oligofructose and ORAFTI P95	Probiotics *vs* prebiotics *vs* placebo	ALT, AST, GGT, HDL-C, LDL-C, TC, TG	MASLD	Non-invasive imaging and serology
Nabavi et al., 2014	72	2	42.75 ± 8.72	44.05 ± 8.14	RCT	Probiotics yogurt (300g/d) (*n* = 36)	Lactobacillus and Streptococcus (4.42 ×10^6^ CFU/g), Bifidobacterium (3.85 ×10^6^ CFU/g)	Probiotics yogurt *vs* conventional yogurt	ALT, AST, HDL-C, LDL-C, TC, TG	MASLD	Non-invasive imaging
Abd El Hamid et al., 2024 (NCT06074094)	50	3	45.72 ± 8.9	46.48 ± 11.60	RCT	Probiotics (1 capsule/d) (*n* = 25)	Lactobacillus (1 × 10^9^ CFU)	Probiotics *vs* placebo	ALT, AST	MASLD	Non-invasive imaging and serology
Sepideh et al., 2016 (IRCT:2012122911920N1)	42	2	42.10 ± 1.99	47.33 ± 2.53	RCT	Probiotics (1g/d) (*n* = 21)	Lactobacillus (1.35 ×10^10^ CFU/g), Bifidobacterium (2.1 × 10^10^ CFU/g), and Streptococcus (3 × 10^8^ CFU/g)	Probiotics *vs* placebo	TNF-α, IL-6	MASLD	Non-invasive imaging
Manzhalii et al., 2017	65	3	44.3 ± 1.5	43.5 ± 1.3	RCT	Probiotics (1 capsule/d) (*n* = 38)	Lactobacillus, Bifidobacterium, and Streptococcus (1 × 10^8^ CFU /capsule)	Probiotics *vs* placebo	ALT, AST, GGT, TC, TG, LSM	MASLD (MASH)	Non-invasive imaging and serology
Sayari, Neishaboori & Jameshorani, 2018	138	4	42.48 ± 11.41	43.42 ± 11.65	RCT	Synbiotics (0.5g/d) (*n* = 70)	Lactobacillus, Bifidobacterium, Streptococcus (1 × 10^9^ CFU), and fructooligosaccharide	Synbiotics and Sitagliptin *vs* placebo and Sitagliptin	ALT, AST, TC, TG, HDL-C, LDL-C	MASLD	Non-invasive imaging and serology
Eslamparast et al., 2014	52	7	46.35 ± 8.8	45.69 ± 9.5	RCT	Synbiotics (2 capsules/d) (*n* = 26)	Lactobacillus, Bifidobacterium, Streptococcus (2 × 10^8^ CFU), and fructooligosaccharide	Synbiotics *vs* placebo	ALT, AST, GGT, TNF-α	MASLD	Non-invasive imaging and serology
Mofidi et al., 2017 (NCT02530138)	42	7	40.09 ± 11.44	44.61 ± 10.12	RCT	Synbiotics (2 capsule/d) (*n* = 21)	Lactobacillus, Bifidobacterium, Streptococcus (2 × 10^8^ CFU), and fructo-oligosaccharide	Synbiotics *vs* placebo	ALT, AST, GGT, HDL-C, LDL-C, TC, LSM, CAP, TNF-α	MASLD	Non-invasive imaging and serology
Reshef et al., 2024 (NCT02642172)	19	3	47.8 ± 10.37	50 ± 14.52	RCT	Prebiotics (1 sachet/day in the first week and 2 sachets/d thereafter) (*n* = 8)	NA	Prebiotics *vs* placebo	ALT, AST, GGT, HDL-C, LDL-C, TC, TG	MASLD	Non-invasive imaging and serology
Chong et al., 2020 (ANZCTR: 12613001002774)	40	3	50.6 ± 10.4	46.7 ± 11.2	RCT	Antibiotics combination (metronidazole 0.4 g bid for 7 days and inulin 4 g for 3 months) (*n* = 20)	NA	Antibiotics combination *vs* placebo	ALT, AST, GGT, HDL-C, LDL-C, TC, TG, CAP	MASLD	Liver biopsy, non-invasive imaging and serology
Ferolla et al., 2016	50	3	NA	NA	RCT	Synbiotics (10 g/d) (*n* = 27)	Lactobacillus (1 × 10^8^ CFU), guar gum, and inulin	Synbiotics *vs* placebo	ALT, AST, GGT, HDL-C, LDL-C, TC, TG	MASLD (MASH)	Liver biopsy
Abdel-Razik et al., 2018	50	6	40.2 ± 9.88	38.4 ± 9.21	RCT	Antibiotics (rifaximin 1.1g/d)(*n* = 25)	NA	Antibiotics vs placebo	ALT, AST, GGT, TC, TG, TNF-α, IL-6	MASLD (MASH)	Liver biopsy and serology
Fogacci et al., 2024	50	3	61 ± 4	60 ± 5	RCT	Postbiotics (calcium butyrate 0.5g/tablet, zinc gluconate 0.005g/tablet, and vitamin D3 500 IU/tablet, 2 tablets/d) (*n* = 25)	NA	Postbiotics *vs* placebo	HDL-C, LDL-C, TC, TG	MASLD	Non-invasive imaging
Savytska et al., 2025 (NCT06352697)	52	3	NA	NA	RCT	Postbiotic/ metabiotics (200 mg/d)(*n* = 26)	NA	Postbiotics vs placebo	ALT, AST	MASLD	NA
Won et al., 2025 (NCT04555434.)	110	2	NA	NA	RCT	Probiotics (3 capsules/d)(*n* = 85)	Lactobacillus (9 × 10^9^ CFU/day)	Probiotics *vs* placebo	ALT, AST, GGT, TC	MASLD	Serology
Yazdani et al., 2025 (IRCT20240212060977N1)	40	3	56.75(26-75)	55.5(37-70)	RCT	Synbiotics (1 capsule/d) (*n* = 20)	Lactobacillus and Bifidobacteriumi (1 × 10^9^ CFU/day), and fructooligosaccharides	Synbiotics *vs* placebo	ALT, AST, HDL-C, LDL-C, TC, TG	MASLD	Non-invasive imaging
Mohammadi et al., 2025 (IRCT20170916036204N6)	80	2	42.25 ± 10.44	43.50 ± 11.00	RCT	Probiotics (500 cc/d) (*n* = 40)	Bifidobacterium and Lactobacillus (2 × 10^9^ CFU)	Probiotics + Diet *vs* Diet	ALT, AST, HDL-C, LDL-C, TC, TG	MASLD	Non-invasive imaging

**Notes.**

Introduction of index abbreviations MASLD metabolic dysfunction-associated steatotic liver disease MASH metabolic dysfunction-associated steatohepatitis T2DM type 2 diabetes mellitus RCT randomized controlled trial ALT alanine aminotransferase AST aspartate aminotransferase GGT gamma-glutamyl transferase CAP controlled attenuation parameter TC total cholesterol TG triglycerides HDL-C high-density lipoprotein cholesterol LDL-C low-density lipoprotein cholesterol TNF-α tumor necrosis factor alpha IL-6 interleukin 6

A comprehensive comparison of key effect modifiers across the treatment arms is provided in Supplemental Information 2 (‘Table S4’).

### Risk of bias

**Figure 3:**
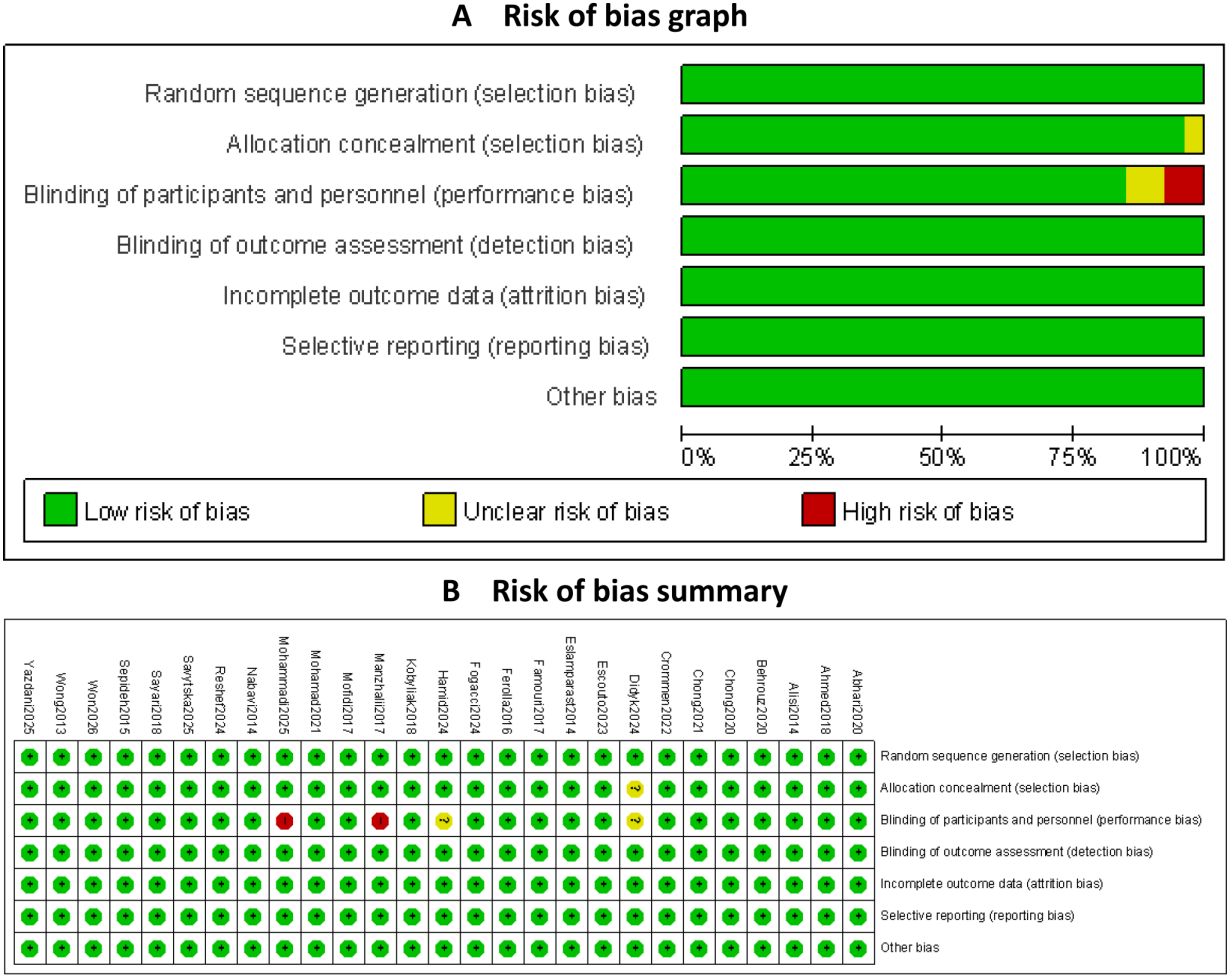
The risk of bias of all included RCTs.

The risk of bias of all included RCTs is summarized in Fig. 3. As shown in Fig. 3A, the majority of trials were judged to have a low risk of bias across most domains. The overall distribution of risk of bias across studies is presented in Fig. 3B, indicating that most RCTs were of generally good methodological quality. In the risk of bias assessment, performance bias in studies (Didyk et al., 2024) and (Abd El Hamid et al., 2024), along with allocation concealment in study (Didyk et al., 2024), were rated as “unclear risk” due to the lack of explicit methodological descriptions. Two studies (Manzhalii et al., 2017) and (Mohammadi et al., 2025) were assessed as having a “high risk” of performance bias because the authors explicitly acknowledged the absence of blinding in their trials. The methodological quality across the majority of trials remained consistent, providing a basis for the synthesis of the evidence. A comprehensive assessment of the risk of bias for all included studies is detailed in Supplemental Information 2 (‘Table S7’), including specific justifications for all domains classified as having an unclear or high risk of bias.

### Heterogeneity analysis

The findings suggest that the overall heterogeneity of the study is minimal. The global inconsistency test indicated good consistency (*P* > 0.05) (Supplemental Information 2, p1). Node inconsistency analysis revealed no significant discrepancies between the local direct and indirect comparison evidence, with all *P*-values exceeding 0.05 (Supplemental Information 2, p1). Additionally, the loop inconsistency analysis confirmed the absence of significant inconsistencies within the loop (*P* = 0.778) (Supplemental Information 2, p1). Consequently, the evidence network constructed in this study supports the consistency hypothesis.

### Effect of gut microbiota-regulating drugs on liver enzymes

#### Effect of gut microbiota-regulating drugs on ALT

**Figure 4:**
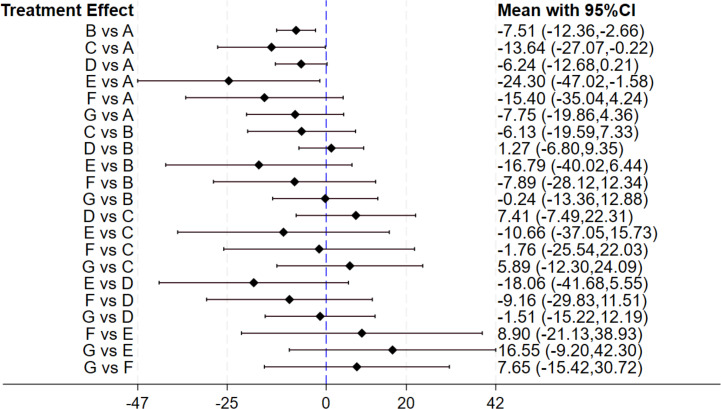
The forest plot results of ALT. Forest plot of pairwise comparisons between interventions. Diamonds indicate MD with corresponding 95% CI. The unit of measurement for the outcome variable is U/L. Values greater than 0 indicate increased ALT levels, whereas values below 0 reflect decreases.

A total of 25 studies examined the impact of gut microbiota-regulating drugs on ALT levels. Among these, 14 investigated probiotics, five focused on synbiotics, and two on prebiotics. Of all the studies, 16 had an intervention duration of 12 weeks or fewer, while nine extended beyond 12 weeks. The forest plot (Fig. 4) revealed that compared with placebo, statistically significant differences were identified in groups probiotics (MD: −7.51, 95% CI [−12.36 to −2.66]), prebiotics (MD: −13.64, 95% CI [−27.07 to −0.22]), and antibiotics (MD: −24.30, 95% CI [−47.20 to −1.58]), while no statistically significant differences were noticed in other comparisons among interventions. The SUCRA ranking results (Table 2) showed that the top three interventions were antibiotics (86.7), postbiotics (68.8), prebiotics (66.8), with placebo ranked the lowest (3.9). The ranking graph confirms that antibiotics and prebiotics are ranked higher than the placebo group in terms of efficacy. Although antibiotics ranked first, it is important to note that the SUCRA ranking does not account for statistical differences. Therefore, there were no statistically significant differences in the improvement of ALT levels among the three interventions.

**Table 2:** The SUCRA ranking of primary outcomes.

Treatment	Outcomes	SUCRA	PreBest	MeanRank
A	ALT	3.9	0	6.8
B		43.2	0.2	4.4
C		66.8	12.1	3
D		36.6	0.3	4.8
E		86.7	62.4	1.8
F		68.8	22.4	2.9
G		44	2.6	4.4
A	AST	12	0	6.3
B		46.3	0.8	4.2
C		34.4	3.6	4.9
D		77.9	24.3	2.3
E		69.7	40.7	2.8
F		50.8	16.7	3.9
G		59	13.9	3.5
A	GGT	13	0	5.3
B		24.3	0.1	4.8
C		60.1	16.3	3
D		63	9.2	2.9
E		72.5	38.9	2.4
F		67.1	35.4	2.6
A	CAP	26.8	0	3.2
B		51.1	1.1	2.5
D		99.5	98.4	1
G		22.6	0.5	3.3
A	LDL-C	24.9	0	4.8
B		42.5	0.6	3.9
C		79.7	41.9	2
D		73.3	20.5	2.3
E		70.5	36	2.5
F		9.1	1	5.5

**Notes.**

Introduction of index abbreviations ALT alanine aminotransferase AST aspartate aminotransferase GGT gamma-glutamyl transferase CAP controlled attenuation parameter LDL-C low-density lipoprotein cholesterol

Intervention abbreviations A placebo or usual care controls B probiotics C prebiotics D synbiotics E antibiotics F postbiotics G combination of antibiotics and gut microbiota-regulating drugs (combination treatment)

SUCRA rankings are based on the magnitude of cumulative ranking probabilities. A higher SUCRA value indicates a higher probability of being the best treatment but does not imply statistically significant pairwise superiority over interventions with lower rankings.

#### Effect of gut microbiota-regulating drugs on AST

**Figure 5:**
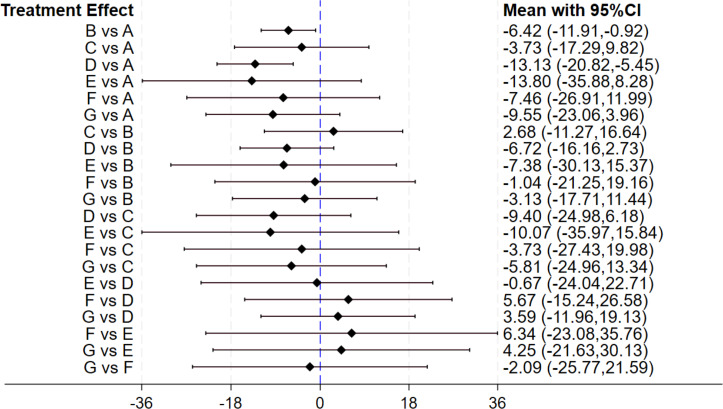
The forest plot results of AST. The outcome measure is expressed in units of U/L. A value greater than 0 indicates an increase in AST levels, whereas a value less than 0 indicates a decrease in AST levels.

Twenty four studies assessed the impact of gut microbiota-regulating drugs on AST levels. Of these, 13 evaluated probiotics, two prebiotics, five synbiotics, one antibiotic, and two combination treatment. Sixteen studies had a duration of 12 weeks or less, and eight exceeded this duration. The forest plot (Fig. 5) demonstrated that, in comparison with the control group, both probiotics (MD: −6.42, 95% CI [−11.91 to −0.92]) and synbiotics (MD: −13.13, 95% CI [−20.82 to −5.45]) led to a significant improvement in AST levels. No statistically significant variances were observed in pairwise comparisons among the other interventions. The SUCRA ranking results (Table 2) indicated that synbiotics (77.9) ranked first, followed by antibiotics (69.7) in second place, and combination treatment (59) in third, with placebo ranked last. However, the forest plot results reveal no statistically significant differences between the interventions, thus there is no notable difference in their effect on reducing AST levels.

#### Effect of gut microbiota-regulating drugs on GGT

**Figure 6:**
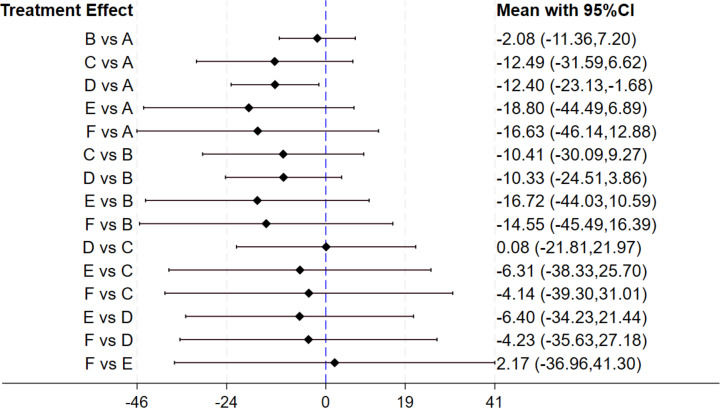
The forest plot results of GGT. The outcome metric is presented in U/L. Values greater than 0 reflect elevated GGT levels, while values below 0 represent reductions in GGT levels.

Thirteen studies investigated the therapeutic effects on GGT levels. Among these, six studies evaluated probiotics, two prebiotics, and four synbiotics. Nine studies had an intervention period of 12 weeks or less, and four exceeded this duration. The forest plot (Fig. 6) revealed that synbiotics exerted the significant impact on enhancing GGT levels (MD: −12.40, 95% CI [−23.13 to −1.68]). The SUCRA ranking results (Table 2) illustrated that, in terms of improving GGT levels in MASLD patients, antibiotics (72.5) ranked highest, followed by postbiotics (67.1), synbiotics (63), prebiotics (60.1), and probiotics (24.3), with placebo (13) showing the least improvement. While synbiotics demonstrated a greater reduction in GGT levels compared to placebo, the forest plot analysis revealed no statistically significant differences between synbiotics and the other interventions.

#### Effect of gut microbiota-regulating drugs on CAP

**Figure 7:**
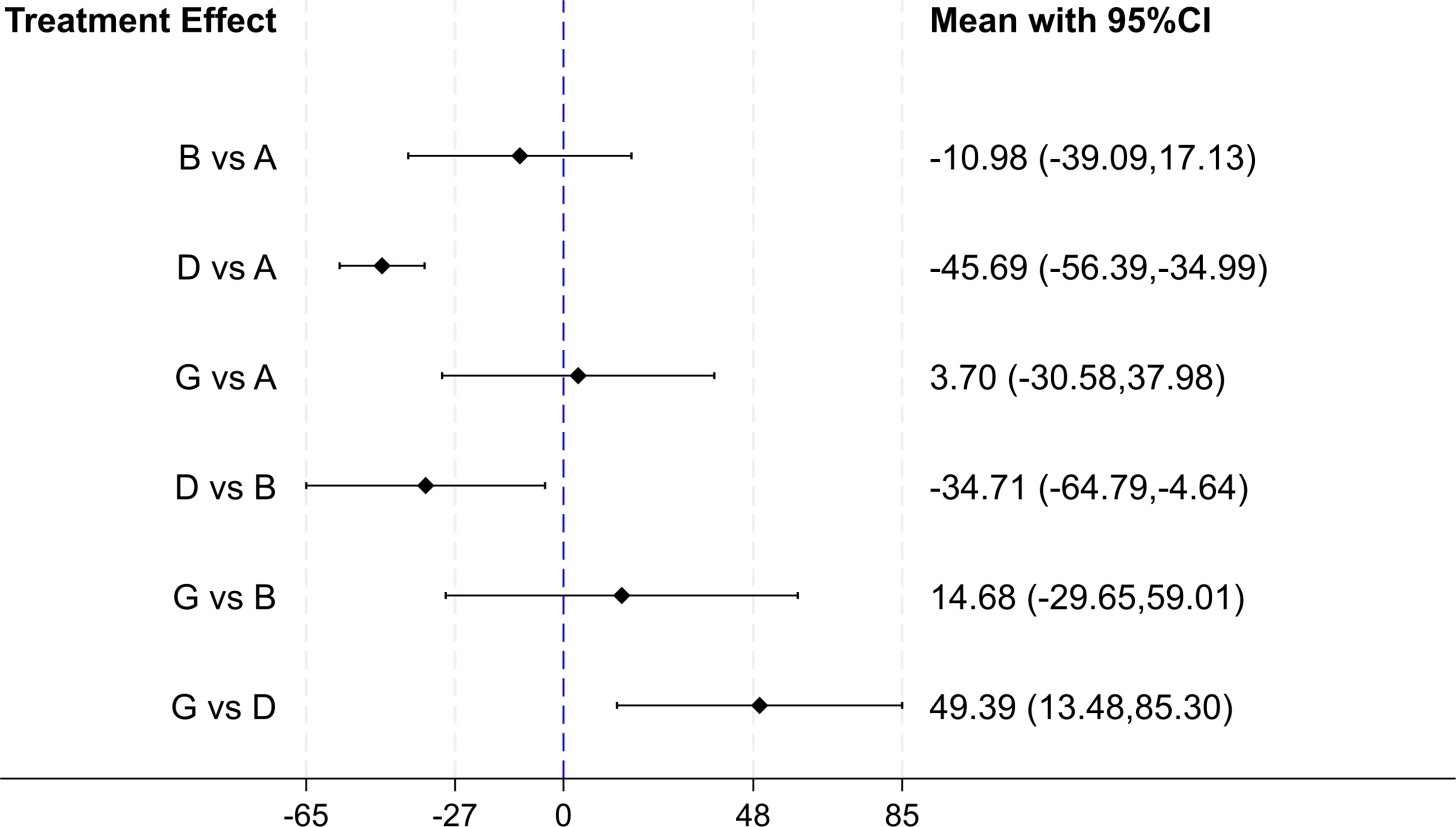
The forest plot results of CAP. Outcome values were reported in dB/m. A value above 0 denotes a CAP increase, whereas a value below 0 denotes a CAP decrease.

Four studies examined the effect of interventions on hepatic steatosis. Among these, two studies focused on synbiotics, one on probiotics, and one on antibiotics. Two studies had an intervention duration of 12 weeks, while the other two lasted more than 12 weeks. The results of forest plot (Fig. 7) indicated that synbiotics demonstrated a substantial improvement in CAP comparison to placebo (MD: −45.69, 95% CI [−56.39 to −34.99]), probiotics (MD: −34.71, 95% CI [−64.79 to −4.64]) and antibiotics combined with gut microbiota-regulating drugs (MD: 49.39, 95% CI [13.48–85.30]). The SUCRA ranking results (Table 2) revealed that synbiotics (99.5) were the most effective in enhancing the CAP levels of patients with MASLD, followed by probiotics (51.1) in second place. Based on the combined findings from the forest plot and SUCRA rankings, synbiotics may be considered the most effective treatment for improving CAP in the studies included in this meta-analysis.

Given that only four trials contributed to the CAP outcome, we performed a leave-one-out sensitivity analysis to evaluate the robustness of the SUCRA-based ranking. The analysis showed minimal variation in SUCRA values across iterations (all SD < 1%), suggesting that the treatment hierarchy was stable and not driven by any single study (Supplemental Information 2 (‘Table S5’)).

### Effect of gut microbiota-regulating drugs on lipid profiles

**Figure 8:**
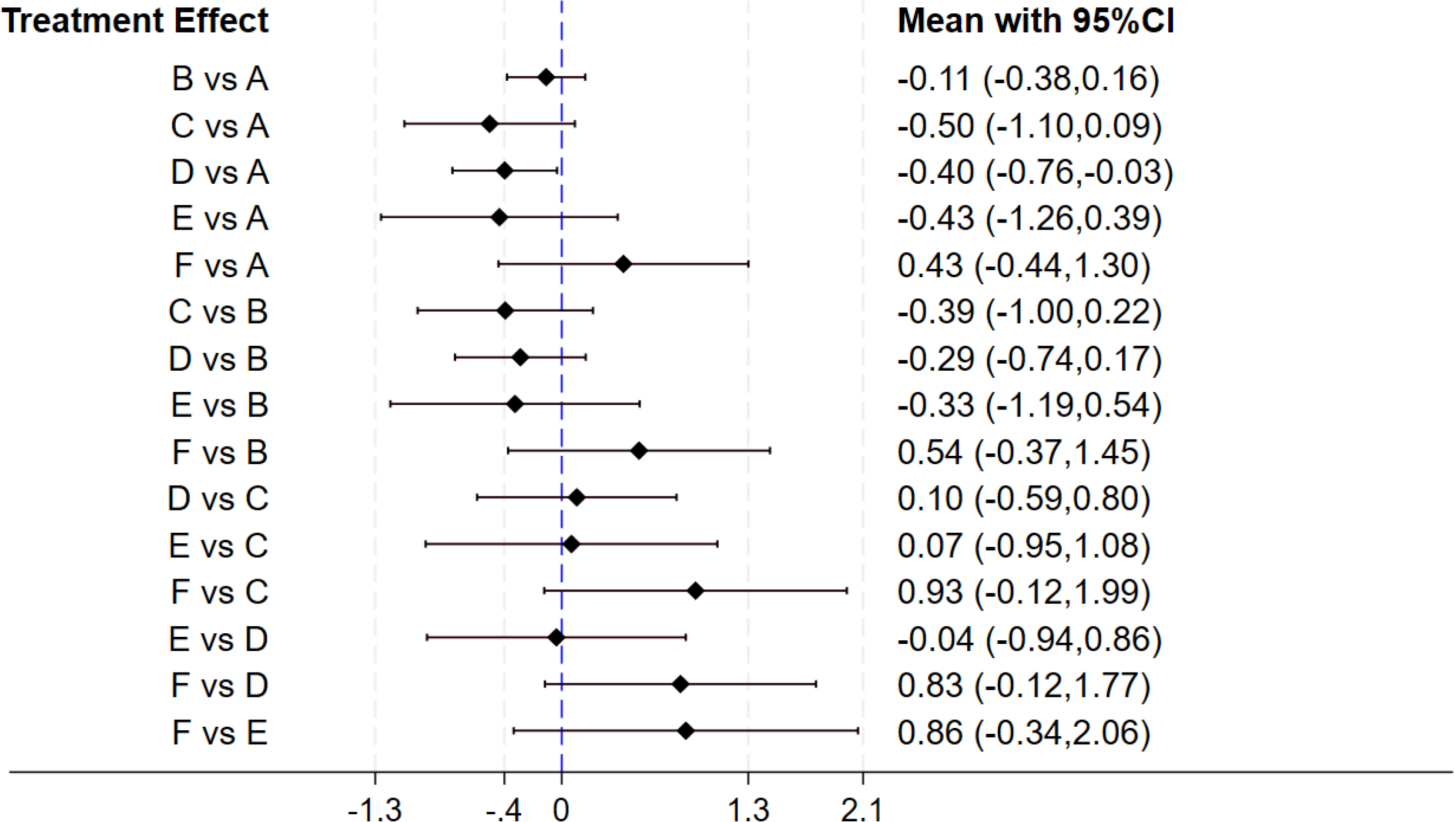
The forest plot results of LDL-C. The outcome metric is presented in mg/dL or mmol/L. Values greater than 0 reflect elevated LDL-C levels, while values below 0 represent reductions in LDL-C levels.

Seventeen studies investigated the therapeutic effects on LDL-C levels. Among these, nine studies evaluated probiotics, two prebiotics, and fur synbiotics. Thirteen studies had a duration of 12 weeks or less, and four exceeded this duration. The forest plot (Fig. 8) revealed that synbiotics exerted the significant impact on improving LDL-C levels (MD: −0.40, 95% CI [−0.76 to −0.03]). The SUCRA ranking results (Table 2) illustrated that prebiotics (79.7) ranked highest, followed by synbiotics (73.3), antibiocits (70.5), and probiotics (42.5), with combination treatment (9.1) showing the least improvement. No statistically significant differences were observed between synbiotics and other active interventions.

Data regarding TC, TG, and HDL-C were reported in 21, 20, and 17 studies, respectively, with interventions primarily focused on probiotics and synbiotics. The forest plot results show no statistically significant differences between any of the interventions and placebo in terms of improving lipid profiles (Supplemental Information 2, p21–p23).

### Effect of gut microbiota-regulating drugs on TNF-α and IL-6

Seven studies assessed the therapeutic effect on TNF-α levels and five studies evaluated the effect of interventions on IL-6 levels, with probiotics and antibiotics as the primary interventions. The forest plot indicates that, compared to placebo, none of the interventions showed statistically significant differences in improving TNF-α or IL-6 levels (Supplemental Information 2, p24–p25).

### Meta-regression analysis

Our analysis demonstrated that probiotics and synbiotics significantly improved ALT, AST, GGT, CAP, and LDL-C levels. Subsequently, we performed a meta-regression analysis to evaluate whether treatment duration influenced these outcomes. The results indicated that treatment duration was not a significant moderator, as the 95% CIs for all beta coefficients encompassed zero (Supplemental Information 2, p20).

### Small-study effect analysis

**Figure 9:**
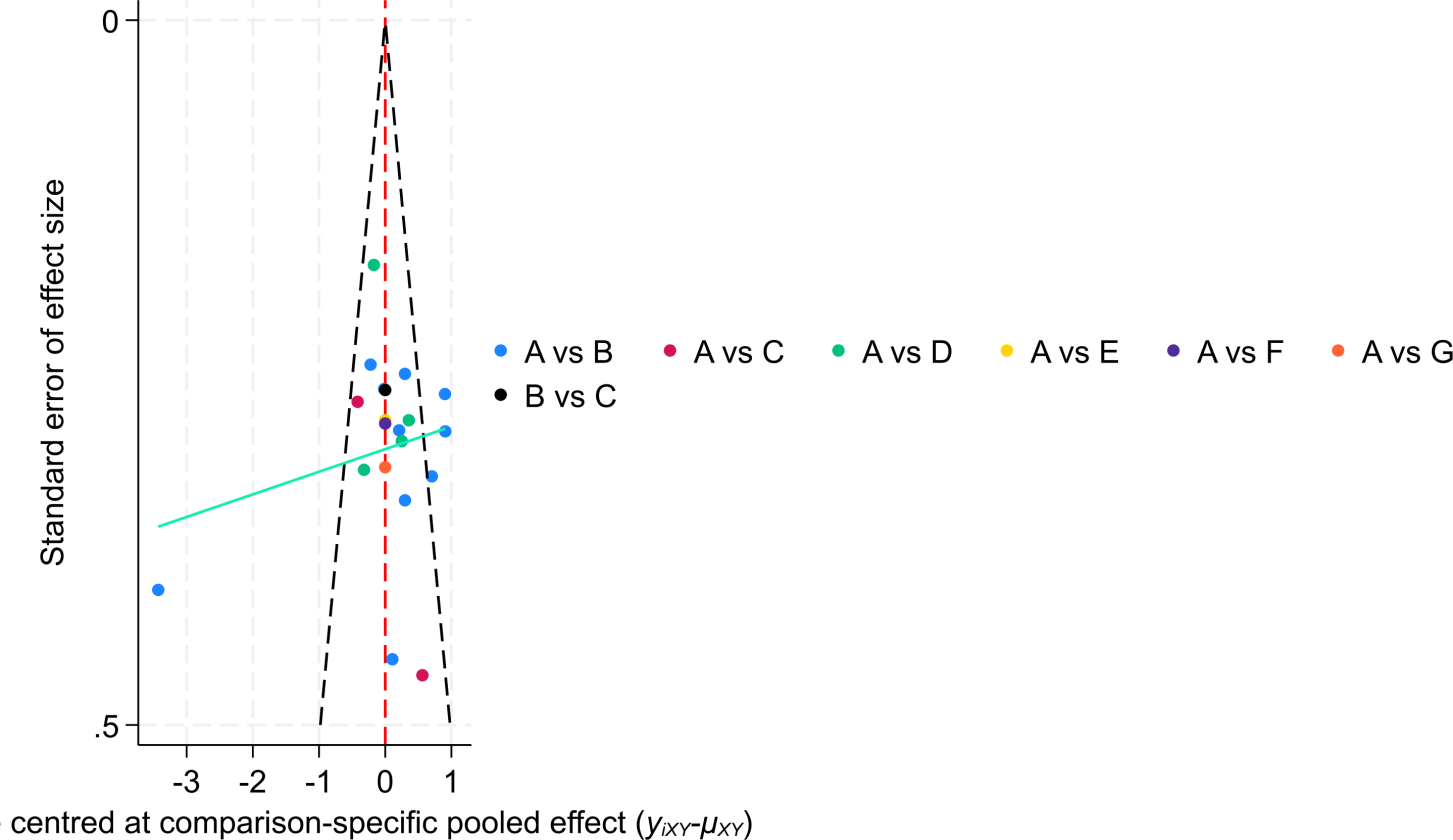
The funnel plot of the study.

The results of the comparison-adjusted funnel plots suggested that there may not be small-study effects for efficacy and safety (Fig. 9).

### Certainty of evidence assessment

CINeMA assessment indicated an overall moderate level of certainty across most comparisons. The evidence was generally consistent, with low risk of reporting bias and no major concerns regarding indirectness or incoherence. Comparisons *versus* placebo showed moderate confidence, suggesting a reasonably robust evidence base for the beneficial effects of gut microbiota-regulating drugs. Detailed results are provided in Table 3.

**Table 3:** Certainty of evidence of the study.

Comparison	Within-study bias	Reporting bias	Indirectness	Imprecision	Heterogeneity	Incoherence	Confidence rating
A:B	Some concerns	Low risk	No concerns	No concerns	Some concerns	No concerns	Moderate
A:C	Some concerns	Low risk	No concerns	Some concerns	Some concerns	No concerns	Moderate
A:D	Some concerns	Low risk	No concerns	No concerns	Some concerns	No concerns	Moderate
A:E	Some concerns	Low risk	No concerns	Some concerns	Some concerns	No concerns	Moderate
B:D	Some concerns	Low risk	No concerns	Some concerns	Some concerns	No concerns	Low
B:G	Some concerns	Low risk	No concerns	Some concerns	Some concerns	No concerns	Low

**Notes.**

Intervention abbreviations A placebo or usual care controls B probiotics C prebiotics D synbiotics E antibiotics F postbiotics G combination of antibiotics and gut microbiota-regulating drugs (combination treatment)

## Discussion

The development and progression of MASLD are closely associated with alterations in the gut microbiota composition. Disruptions in the bidirectional gut-liver axis during ecological dysbiosis compromise gut barrier integrity, facilitating the relocation of microbial antigens and metabolic products to the liver. This triggers hepatic responses, including the secretion of antibodies and bile acids into the gut, providing a theoretical model for understanding the role of gut dysbiosis in MASLD pathogenesis and informing the development of microbiota-targeted therapies. However, the optimal therapeutic strategy for modulating gut microbiota in MASLD remains undetermined, with no established consensus on the most effective approach (Chong et al., 2020;). To address this, a systematic review and network meta-analysis were conducted to evaluate potential treatments.

This analysis included 27 RCTs examining the effectiveness of pharmacological interventions aimed at intestinal flora in MASLD. Both probiotics and synbiotics demonstrated significant improvements in primary clinical outcomes. The majority of studies employed intervention durations of approximately 12 weeks (Famouri et al., 2017; Crommen et al., 2022; Abhari et al., 2020; Behrouz et al., 2020; Abd El Hamid et al., 2024; Manzhalii et al., 2017; Reshef et al., 2024; Chong et al., 2020; Ferolla et al., 2016; Fogacci et al., 2024; Savytska et al., 2025; Yazdani et al., 2025), leading to the hypothesis that a 12-week treatment period may be the threshold for achieving therapeutic efficacy. While some studies suggest that longer intervention durations may enhance outcomes, the benefits do not appear to scale linearly with extended durations.

Liver function improvement in MASLD patients was evaluated using standard clinical biomarkers, including elevated serum levels of ALT, AST and GGT, which act as dependable markers of damage to the liver. Hepatic steatosis was quantified using the CAP *via* the FibroScan system (Echosens, Paris, France), which assesses ultrasound attenuation correlated with liver fat content. The meta-analysis revealed that probiotics, prebiotics, and antibiotics significantly reduced ALT levels, while probiotics and synbiotics significantly decreased AST levels, and synbiotics also reduced GGT levels. These findings aligned with previous studies, such as the network meta-analysis by Kanchanasurakit et al. (2022) and the clinical trial by Mantri et al. (2024), which demonstrated that probiotic, prebiotics and synbiotic supplementation effectively mitigated disease progression and improved liver enzyme profiles. The reduction in liver enzyme levels carries significant clinical implications for both disease prognosis and the improvement of MASLD pathology. Notably, even marginal reductions in these biochemical markers are associated with meaningful gains in long-term clinical outcomes. Furthermore, synbiotics provided significant improvements in hepatic steatosis compared to other interventions, corroborating prior research (Rong et al., 2023).

In this meta-analysis, synbiotics were found to reduce LDL-C levels, a finding consistent with previous research (Kanchanasurakit et al., 2022). Conversely, no significant impact was observed on other lipid parameters. This lack of effect aligns with prior studies (Xiao et al., 2019; Musazadeh et al., 2024), which similarly reported no substantial alterations in lipid markers such as TC, TG and HDL-C. However, other meta-analyses, such as one involving 24 studies with 1,403 MASLD patients, suggested that probiotics significantly reduced TG and HDL-C levels (Huang et al., 2022). The variability in outcomes may stem from confounding factors, including specific probiotic strains and treatment duration, which could differentially influence lipid metabolism in MASLD patients (Huang et al., 2022).

The pathogenesis of MASLD involves a vicious cycle of lipotoxicity, steatosis, and inflammation, with nuclear factor κB (NF-κB) activation playing a pivotal role in driving inflammatory responses *via* cytokines such as TNF-α and IL-6. This meta-analysis did not observe significant reductions in TNF-α or IL-6 levels with probiotic use, consistent with findings by Loman et al. (2018). However, other studies reported significant reductions in these cytokines after 8 weeks of probiotic supplementation (Ahn et al., 2019), suggesting that small sample sizes or other methodological differences may account for discrepancies.

Probiotics are live microorganisms that supply positive health effects, while prebiotics are fermentable dietary fibers that modulate gut microbiota activity. Synbiotics combine both, offering synergistic effects (Liu et al., 2019). Probiotic interventions, particularly those containing Lactobacillus and Bifidobacterium strains, are the most extensively studied approach for MASLD treatment. Despite promising findings, significant heterogeneity exists regarding the efficacy of different probiotic regimens, potentially reflecting variations in clinical phenotypes and microbial compositions associated with MASLD (Sharpton, Ajmera & Loomba, 2019). Overlap in the formulations of probiotics and synbiotics used in included studies further complicates direct comparisons.

Overall, our findings align with previous evidence suggesting that synbiotics may confer clinical benefits in the management of MASLD. The improvement in CAP observed with synbiotic supplementation in this study is consistent with prior literature (Xiao et al., 2019), in which reductions in liver enzymes are commonly reported and decreases in hepatic fat content have been noted in some trials. These parallels support the biological plausibility that modulating the gut microbiota may ameliorate hepatic steatosis, although larger studies are needed to confirm the robustness of this effect. It is important to note that efficacy likely varies by probiotic strain and dosage. Given the lack of standardization in current trials, strain-specific analyses were not feasible in this review. We emphasize the need for future rigorous, standardized RCTs to elucidate the therapeutic potential of specific strains and dosages for MASLD.

Our results indicate that antibiotics confer no significant improvement in key prognostic indicators for MASLD. Therefore, antibiotic therapy is unjustified in non-infected MASLD patients and should be avoided in the absence of concurrent infection.

### Limitations

There are several limitations to this meta-analysis. As we did not search the Scopus and ScienceDirect databases, some relevant studies may have been overlooked. We acknowledge the potential for publication bias, particularly in smaller studies where positive results may be more likely to be reported, leading to potential bias. These smaller studies may be overrepresented in the analysis, which could affect the generalizability of the findings. The limited number of concurrent studies made it difficult to perform subgroup analyses of hepatic steatosis and fibrosis. In addition, the efficacy of synbiotics in reducing CAP was based on only four trials. Although synbiotics ranked first in SUCRA, this ranking does not imply statistical significance. Further clinical studies are warranted to validate this finding. Furthermore, many trials lacked sequential liver biopsy or advanced imaging techniques, such as magnetic resonance imaging-derived proton-density fat fraction (MRI-PDFF), to assess hepatic steatosis. While serum biomarkers are more clinically accessible, liver biopsy and imaging remain the gold standards for the precise assessment of hepatic inflammation and steatosis. Additionally, the characteristics of probiotic and synbiotic therapies, including varying strain combinations and dosages, remain undefined.

## Conclusions

Antibiotics do not significantly reduce hepatic steatosis, and their routine use is not recommended for non-infected MASLD patients. This network meta-analysis demonstrates that probiotics and synbiotics effectly improve liver enzymes, with synbiotics also showing potential in reducing CAP levels. However, evidence supporting synbiotics in reducing CAP is limited by the small number of included studies. Therefore, accumulating more data is necessary to confirm this specific effect. Future large-scale RCTs utilizing standardized strains, dosages, and gold-standard diagnostics such as MRI-PDFF or liver biopsy, are essential to validate these outcomes.

## Supplemental Information

10.7717/peerj.21166/supp-1 Supplemental Information 1 Intended Audience

10.7717/peerj.21166/supp-2 Supplemental Information 2 Supplemental figures and tables

10.7717/peerj.21166/supp-3 Supplemental Information 3 PRISMA checklist The PRISMA (Preferred Reporting Items for Systematic Reviews and Meta-Analyses) checklist is a set of guidelines designed to help authors improve the reporting of systematic reviews and meta-analyses. The checklist consists of 27 essential items that should be included when reporting on a systematic review or meta-analysis to ensure transparency and consistency.

10.7717/peerj.21166/supp-4 Supplemental Information 4 Raw data

10.7717/peerj.21166/supp-5 Supplemental Information 5 Stata code

## Additional Information and Declarations

### Data Availability

The following information was supplied regarding data availability: This is a systematic review/meta-analysis.
