# Supplementary material for: The efficacy of gut microbiota-regulating drugs on metabolic dysfunction-associated steatotic liver disease: a systematic review and network meta-analysis
Source: PeerJ. 2026 Apr 20;14:e21166. doi: 10.7717/peerj.21166 (PMC13105187; doi:10.7717/peerj.21166)
Supplement: Supplemental Information 1 [file peerj-14-21166-s001.docx]

**Intended Audience**

The primary audience for this systematic review and network meta-analysis includes clinical practitioners and researchers in the fields of gastroenterology and hepatology. The findings are intended to assist clinicians in optimizing therapeutic strategies for the management of metabolic dysfunction-associated steatotic liver disease (MASLD) by offering a comparative evaluation of gut microbiota-modulating agents. Additionally, this study aims to support medical researchers by providing a comprehensive synthesis of current evidence to inform future clinical research and guideline development.
