# Supplementary material for: The efficacy of gut microbiota-regulating drugs on metabolic dysfunction-associated steatotic liver disease: a systematic review and network meta-analysis
Source: PeerJ. 2026 Apr 20;14:e21166. doi: 10.7717/peerj.21166 (PMC13105187; doi:10.7717/peerj.21166)
Supplement: Supplemental Information 2 [file peerj-14-21166-s002.docx]

**Supplementary appendix**

Supplementary Tabel 1 Global inconsistency analysis of network meta-analysis

|  |  | **Coefficient** | **Std.err.** | **z** | **P>\|z\|** | **[95%conf.interval]** | |
| --- | --- | --- | --- | --- | --- | --- | --- |
| **_y_B** |  |  |  |  |  |  |  |
|  | des_ABC | -0.01265 | 1.149419 | -0.01 | 0.991 | -2.26547 | 2.240169 |
|  | _cons | -0.53358 | 0.368454 | -1.45 | 0.148 | -1.25574 | 0.188577 |
| **_y_C** |  |  |  |  |  |  |  |
|  | des_AC | -0.97874 | 1.588437 | 0.62 | 0.538 | -2.13454 | 4.09202 |
|  | _cons | -0.95894 | 1.090982 | -0.88 | 0.379 | -3.09722 | 1.179374 |
| **_y_D** |  |  |  |  |  |  |  |
|  | _cons | -0.35782 | 0.545832 | -0.66 | 0.512 | -1.42763 | -0.71199 |
| **_y_E** |  |  |  |  |  |  |  |
|  | _cons | -0.21773 | 1.094295 | -0.2 | 0.842 | -2.36251 | 1.927044 |
| **_y_F** |  |  |  |  |  |  |  |
|  | _cons | -0.41606 | 1.094915 | -0.38 | 0.704 | -2.56205 | 1.729938 |
| **_y_G** |  |  |  |  |  |  |  |
|  | _cons | 0.201383 | 1.103416 | 0.18 | 0.855 | -1.96127 | 2.364039 |

Supplementary Tabel 2 Node inconsistency analysis of network meta-analysis

| **Side** | | **Direct Coef.** | **Std.Err.** | **Indirect Coef.** | **Std.Err.** | **Difference Coef.** | **Std.Err.** | **P>\|z\|** | **tau** |
| --- | --- | --- | --- | --- | --- | --- | --- | --- | --- |
|  |  |  |  |  |  |  |  |  |  |
| **A** | **B** | -0.533 | 0.33255 | 1.411196 | 2.84427 | -1.9442 | 2.86793 | 0.498 | 1.002432 |
| **A** | **C** | -0.49876 | 0.772029 | -0.93112 | 1.967083 | 0.432373 | 2.111367 | 0.838 | 1.025392 |
| **A** | **G** | -0.41168 | 1.084477 | 0.314017 | 1.051111 | -0.7257 | 1.48379 | 0.625 | 1.015127 |

Supplementary Tabel 3 Loop inconsistency analysis of network meta-analysis

| **Loop** | **If** | **self** | **z_value** | **p_value** | **CI_95** | **Loop_Heterog_tau2** |
| --- | --- | --- | --- | --- | --- | --- |
| **A-B-C** | 0.444 | 1.58 | 0.281 | 0.779 | (0.00,3.54) | 0.992 |

Supplementary Tabel 4 Baseline of studies included population

| **Author /year** | **Mohamad/2021** | | **Didyk/2024** | | **Escouto/2023** | | **Famouri/2017** | | **Kobyliak/2018** | |
| --- | --- | --- | --- | --- | --- | --- | --- | --- | --- | --- |
| **characteristic** | **probiotics** | **placebo** | **metformin and antibiotics combination** | **metformin** | **probiotics** | **placebo** | **probiotics** | **placebo** | **probiotics** | **placebo** |
| **number** | **17** | **22** | **34** | **34** | **23** | **25** | **32** | **32** | **30** | **28** |
| **Age- years ± SD** | **54.70±10.19** | **52.47±16.73** | **59.8±3.4** | **58.6±2.9** | **54.70±10.19** | **52.47±16.73** | **12.7±2.2** | **12.6±1.7** | **53.4±9.55** | **57.29±10.45** |
| **Male (%)** | **11(64.71%)** | **17(77.27)** | **16(47%)** | **14(41%)** | **11(13.0%)** | **7(28.0%)** | **14(43.8%)** | **18(56.2%)** | **NA** | **NA** |
| **BMI (kg/m^2^ )** | **31.33±12.02** | **28.30±3.90** | **31.6±3.93** | **31.7±4.59** | **31.6** | **31.6** | **26.44±4.3** | **26.61±2.26** | **34.82±6.84** | **34.26±6.17** |
| **Diabetes mellitus (%)** | **9(52.94%)** | **10(45.45%)** | **NA** | **NA** | **17(73.9%)** | **19(79.2%)** | **NA** | **NA** | **30(100%)** | **28(100%)** |
| **Hypertension (%)** | **11(64.71%)** | **10(45.45%)** | **NA** | **NA** | **17(73.9%)** | **18(75.0%)** | **NA** | **NA** | **NA** | **NA** |
| **Fibrosis stage F0 (%)** | **NA** | **NA** | **NA** | **NA** | **7 (38.9%)** | **13 (59.1%)** | **NA** | **NA** | **NA** | **NA** |
| **Fibrosis stage F1–F2 (%)** | **NA** | **NA** | **NA** | **NA** | **7 (38.9%)** | **6 (27.3%)** | **NA** | **NA** | **NA** | **NA** |
| **Fibrosis stage F3–F4 (%)** | **NA** | **NA** | **NA** | **NA** | **4 (22.2%)** | **3 (13.6%)** | **NA** | **NA** | **NA** | **NA** |
| **Fatty liver (grade) Grade I (%)** | **NA** | **NA** | **NA** | **NA** | **NA** | **NA** | **20 (62.5)** | **18 (56.2)** | **NA** | **NA** |
| **Fatty liver (grade) Grade II (%)** | **NA** | **NA** | **NA** | **NA** | **NA** | **NA** | **12 (37.5)** | **14 (43.8)** | **NA** | **NA** |
| **Fatty liver (grade) Grade III (%)** | **NA** | **NA** | **NA** | **NA** | **NA** | **NA** | **NA** | **NA** | **NA** | **NA** |

Supplementary Tabel 4 Baseline of studies included population

| **Author /year** | **Chong/2021** | | **Crommen/2022** | | **Wong/2013** | | **Abhari/2020** | | **Alisi/2014** | |
| --- | --- | --- | --- | --- | --- | --- | --- | --- | --- | --- |
| **characteristic** | **probiotics** | **placebo** | **probiotics** | **placebo** | **probiotics** | **placebo** | **synbiotics** | **placebo** | **probiotics** | **placebo** |
| **number** | **19** | **16** | **25** | **23** | **10** | **10** | **22** | **23** | **22** | **22** |
| **Age- years ± SD** | **57± 8** | **58±7** | **40±11** | **41±9** | **42±9** | **55±9** | **47.7±11.4** | **46.7±12.4** | **10±2.22** | **11±1.48** |
| **Male (%)** | **15(78.95%)** | **13(81.25%)** | **6(33%)** | **4 (17%)** | **8(80%)** | **5(50%)** | **14 (61%)** | **11 (50%)** | **10(83.3%)** | **14(63.64%)** |
| **BMI (kg/m^2^ )** | **31.2** | **31.9** | **44.3±3.0** | **43.2±3.4** | **30.2±5.0** | **28.7±5.7** | **32.2±6.72** | **33.6±5.06** | **27.3±2.89** | **25.6±3.49** |
| **Diabetes mellitus (%)** | **15(78.95%)** | **11(68.75%)** | **NA** | **NA** | **3(30%)** | **4(40%)** | **NA** | **NA** | **NA** | **NA** |
| **Hypertension (%)** | **NA** | **NA** | **NA** | **NA** | **3(30%)** | **4(40%)** | **NA** | **NA** | **NA** | **NA** |
| **Fibrosis stage F0 (%)** | **NA** | **NA** | **NA** | **NA** | **3(30%)** | **3(30%)** | **NA** | **NA** | **NA** | **NA** |
| **Fibrosis stage F1–F2 (%)** | **NA** | **NA** | **NA** | **NA** | **6(60%)** | **4(40%)** | **NA** | **NA** | **NA** | **NA** |
| **Fibrosis stage F3–F4 (%)** | **NA** | **NA** | **NA** | **NA** | **1(10%)** | **3(30%)** | **NA** | **NA** | **NA** | **NA** |
| **Fatty liver (grade) Grade I (%)** | **NA** | **NA** | **NA** | **NA** | **0(0%)** | **1(10%)** | **NA** | **NA** | **NA** | **NA** |
| **Fatty liver (grade) Grade II (%)** | **NA** | **NA** | **NA** | **NA** | **5(50%)** | **5(50%)** | **NA** | **NA** | **NA** | **NA** |
| **Fatty liver (grade) Grade III (%)** | **NA** | **NA** | **NA** | **NA** | **5(50%)** | **4(40%)** | **NA** | **NA** | **NA** | **NA** |

Supplementary Tabel 4 Baseline of studies included population

| **Author /year** | **Behrouz/2020** | | | **Nabavi/2014** | | | **Hamid/2024** | | | **Sepideh/2015** | | | **Manzhalii/2017** | | |
| --- | --- | --- | --- | --- | --- | --- | --- | --- | --- | --- | --- | --- | --- | --- | --- |
| **characteristic** | **probiotics** | **prebiotics** | **placebo** | | **probiotics** | **placebo** | | **probiotics** | **placebo** | | **probiotics** | **placebo** | | **probiotics** | **placebo** |
| **number** | **30** | **29** | **30** | | **36** | **36** | | **25** | **25** | | **21** | **21** | | **38** | **37** |
| **Age- years ± SD** | **38.46±7.11** | **38.41±9.21** | **38.43±10.09** | | **42.75±8.72** | **44.05±8.14** | | **45.72 ±8.9** | **46.48 ±11.60** | | **42.10 ± 1.99** | **47.33 ± 2.53** | | **44.3 ± 1.5** | **43.5±1.3** |
| **Male (%)** | **22 (73.3%)** | **20 (69%)** | **21 (70%)** | | **17 (48.2%)** | **18 (50%)** | | **7 (28%)** | **8 (32%)** | | **13 (61.9%)** | **15 (71.4%)** | | **11(28.95%)** | **16(43.24)** |
| **BMI (kg/m^2^ )** | **29.05±3.04** | **29.32±4.36** | **30.12±6.96** | | **30.1±3.61** | **31.4±3.6** | | **33.88±7.43** | **31.21 ±3.32** | | **30.34±1.17** | **29.50±0.84** | | **26.4±0.8** | **26.6±0.7** |
| **Diabetes mellitus (%)** | **NA** | **NA** | **NA** | | **NA** | **NA** | | **NA** | **NA** | | **NA** | **NA** | | **NA** | **NA** |
| **Hypertension (%)** | **NA** | **NA** | **NA** | | **NA** | **NA** | | **NA** | **NA** | | **NA** | **NA** | | **NA** | **NA** |
| **Fibrosis stage F0 (%)** | **NA** | **NA** | **NA** | | **NA** | **NA** | | **NA** | **NA** | | **NA** | **NA** | | **NA** | **NA** |
| **Fibrosis stage F1–F2 (%)** | **NA** | **NA** | **NA** | | **NA** | **NA** | | **NA** | **NA** | | **NA** | **NA** | | **NA** | **NA** |
| **Fibrosis stage F3–F4 (%)** | **NA** | **NA** | **NA** | | **NA** | **NA** | | **NA** | **NA** | | **NA** | **NA** | | **NA** | **NA** |
| **Fatty liver (grade) Grade I (%)** | **NA** | **NA** | **NA** | | **20 (55.6%)** | **21 (58.3%)** | | **NA** | **NA** | | **NA** | **NA** | | **NA** | **NA** |
| **Fatty liver (grade) Grade II (%)** | **NA** | **NA** | **NA** | | **12 (33.3%)** | **12 (33.3%)** | | **NA** | **NA** | | **NA** | **NA** | | **NA** | **NA** |
| **Fatty liver (grade) Grade III (%)** | **NA** | **NA** | **NA** | | **4 (11.1%)** | **3 (8.3%)** | | **NA** | **NA** | | **NA** | **NA** | | **NA** | **NA** |

Supplementary Tabel 4 Baseline of studies included population

| **Author /year** | **Sayari/2018** | | **Eslamparast/2014** | | **Mofidi/2017** | | **Reshef/2024** | | **Chong2020** | |
| --- | --- | --- | --- | --- | --- | --- | --- | --- | --- | --- |
| **characteristic** | **synbiotics** | **placebo** | **synbiotics** | **placebo** | **synbiotics** | **placebo** | **prebiotics** | **placebo** | **antibiotics** | **placebo** |
| **number** | **70** | **68** | **26** | **26** | **21** | **21** | **8** | **11** | **20** | **20** |
| **Age- years ± SD** | **42.48±11.41** | **43.42±11.65** | **46.35±8.8** | **45.69±9.5** | **40.09±11.44** | **44.61±10.12** | **47.8±10.37** | **50±14.52** | **50.6 ±10.4** | **46.7 ±11.2** |
| **Male (%)** | **NA** | **NA** | **14(53.85%)** | **11(42.31%)** | **11(52.38%)** | **12(57.14%)** | **6(75%)** | **9(81.82%)** | **10(50%)** | **13(65%)** |
| **BMI (kg/m^2^ )** | **29.72±3.62** | **29.54±3.71** | **32.1 ± 2.4** | **31.3 ± 2.3** | **23.17±1.01** | **23.20±1.07** | **32.6±2.74** | **32.5±3.63** | **31.4 ±3.4** | **32.6 ±4.3** |
| **Diabetes mellitus (%)** | **NA** | **NA** | **NA** | **NA** | **NA** | **NA** | **0(0%)** | **2(18.18%)** | **7(35%)** | **7(35%)** |
| **Hypertension (%)** | **NA** | **NA** | **NA** | **NA** | **NA** | **NA** | **3(37.5%)** | **4(36.36%)** | **NA** | **NA** |
| **Fibrosis stage F0 (%)** | **NA** | **NA** | **NA** | **NA** | **NA** | **NA** | **NA** | **NA** | **NA** | **NA** |
| **Fibrosis stage F1–F2 (%)** | **NA** | **NA** | **NA** | **NA** | **NA** | **NA** | **NA** | **NA** | **NA** | **NA** |
| **Fibrosis stage F3–F4 (%)** | **NA** | **NA** | **NA** | **NA** | **NA** | **NA** | **NA** | **NA** | **NA** | **NA** |
| **Fatty liver (grade) Grade I (%)** | **NA** | **NA** | **0(0%)** | **0(0%)** | \| **NA** \| **NA** \| \| --- \| --- \| | **NA** | **NA** | **NA** | **NA** | **NA** |
| **Fatty liver (grade) Grade II (%)** | **NA** | **NA** | **17 (65.4)** | **20 (76.9)** | **NA** | **NA** | **NA** | **NA** | **NA** | **NA** |
| **Fatty liver (grade) Grade III (%)** | **NA** | **NA** | **9 (34.6)** | **6 (23.1)** | **NA** | **NA** | **NA** | **NA** | **NA** | **NA** |

Supplementary Tabel 4 Baseline of studies included population

| **Author /year** | **Ferolla/2016** | | **Ahmed/2018** | | **Fogacci2024** | | **Savytska2025** | | **Won2026** | |
| --- | --- | --- | --- | --- | --- | --- | --- | --- | --- | --- |
| **characteristic** | **synbiotics** | **placebo** | **antibiotics** | **placebo** | **postbiotics** | **placebo** | **postbiotics** | **placebo** | **probiotics** | **placebo** |
| **number** | **27** | **23** | **25** | **25** | **25** | **25** | **26** | **26** | **85** | **25** |
| **Age- years ± SD** | **NA** | **NA** | **40.2 ± 9.88** | **38.4 ± 9.21** | **61±4** | **60±5** | **NA** | **NA** | **NA** | **NA** |
| **Male (%)** | **NA** | **NA** | **18(85.71%)** | **16(76.19%)** | **NA** | **NA** | **NA** | **NA** | **NA** | **NA** |
| **BMI (kg/m^2^ )** | **32.5±4.0** | **32.5±4.0** | **33.3±7.45** | **32.8±7.35** | **27.3±2.1** | **27.1±2.2** | **NA** | **NA** | **28.43±4.37** | **29.70±4.32** |
| **Diabetes mellitus (%)** | **10 (45.5%)** | **9 (32.1%)** | **11(44%)** | **12(48%)** | **NA** | **NA** | **NA** | **NA** | **NA** | **NA** |
| **Hypertension (%)** | **19 (82.6%)** | **19 (70.4%)** | **15(60%)** | **14(56%)** | **NA** | **NA** | **NA** | **NA** | **NA** | **NA** |
| **Fibrosis stage F0 (%)** | **10 (43.5%)** | **12 (46.2%)** | **2(8%)** | **2(8%)** | **NA** | **NA** | **NA** | **NA** | **NA** | **NA** |
| **Fibrosis stage F1–F2 (%)** | **10(38.46%)** | **9(39.13%)** | **17(68%)** | **16(64%)** | **NA** | **NA** | **NA** | **NA** | **NA** | **NA** |
| **Fibrosis stage F3–F4 (%)** | **4(15.38%)** | **4(17.39%)** | **6(24%)** | **7(28%)** | **NA** | **NA** | **NA** | **NA** | **NA** | **NA** |
| **Fatty liver (grade) Grade I (%)** | **NA** | **NA** | **9(36%)** | **7(28%)** | **NA** | **NA** | **NA** | **NA** | **NA** | **NA** |
| **Fatty liver (grade) Grade II (%)** | **NA** | **NA** | **13(52%)** | **14(56%)** | **NA** | **NA** | **NA** | **NA** | **NA** | **NA** |
| **Fatty liver (grade) Grade III (%)** | **NA** | **NA** | **3(12%)** | **4(16%)** | **NA** | **NA** | **NA** | **NA** | **NA** | **NA** |

| **Author /year** | **Yazdani2025** | | **Mohammadi2025** | |
| --- | --- | --- | --- | --- |
| **characteristic** | **synbiotics** | **placebo** | **probiotics** | **placebo** |
| **number** | **20** | **20** | **40** | **40** |
| **Age- years ± SD** | **53.63±12.25** | **54.50±8.25** | **42.25±10.44** | **43.50±11.00** |
| **Male (%)** | **3(15%)** | **7(35%)** | **22 (55.0%)** | **13 (32.5%)** |
| **BMI (kg/m^2^ )** | **NA** | **NA** | **NA** | **NA** |
| **Diabetes mellitus (%)** | **NA** | **NA** | **5 (12.5%)** | **4 (10.0%)** |
| **Hypertension (%)** | **NA** | **NA** | **3 (7.5%)** | **3 (7.5%)** |
| **Fibrosis stage F0 (%)** | **NA** | **NA** | **NA** | **NA** |
| **Fibrosis stage F1–F2 (%)** | **NA** | **NA** | **NA** | **NA** |
| **Fibrosis stage F3–F4 (%)** | **NA** | **NA** | **NA** | **NA** |
| **Fatty liver (grade) Grade I (%)** | **NA** | **NA** | **NA** | **NA** |
| **Fatty liver (grade) Grade II (%)** | **NA** | **NA** | **NA** | **NA** |
| **Fatty liver (grade) Grade III (%)** | **NA** | **NA** | **NA** | **NA** |

Supplementary Tabel 5 Sensitivity Analysis of SUCRA Rankings for Treatment Efficacy in CAP

| Treatment | Full Network SUCRA | Leave-One-Out Range | Standard |
| --- | --- | --- | --- |
| Treatment B(probiotics) | 51.10% | 0-1.6% | 0.83% |
| Treatment D(synbiotics) | 99.50% | 98.4-100.0% | 0.75% |
| Treatment G(combination treatment) | 22.60% | 0-0.3% | 0.15% |
| Treatment A(Placebo) | 26.80% | 0% | 0% |

Supplementary Tabel 6 Summary of Adverse events from included studies

| **Adverse Event** | **Placebo/ Control** |  | **Probiotics** | **Prebiotics** | **Synbiotics** | **Antibiotics** | **Postbiotics** | **Combination** | **Notes / Severity** |
| --- | --- | --- | --- | --- | --- | --- | --- | --- | --- |
| **Diarrhea** | 1/28 (3.6%) [Kobyliak2018]  1/16 (6.3%) [Chong2021]  1/24 (4.2%) [Crommen2022]  1/25 (4.0%) [Ahmed2018] |  | 1/30 (3.3%) [Kobyliak2018]  2/85 (2.4%) [Won 2026] | NA | NA | 2/25 (8.0%) [Ahmed2018] | NA | NA | Mostly short-term, mild |
| **Nausea** | 2/25 (8.0%) [Ahmed2018] |  | 2/19 (10.5%) [Chong2021]  1/85 (1.2%) [Won 2026] | NA | NA | 2/25 (8.0%) [Ahmed2018] | NA | NA | Mild |
| **Bloating** | NA |  | 2/19 (10.5%) [Chong2021]  1/85 (1.2%) [Sung2026] | NA | NA | NA | NA | NA | Mild |
| **Abdominal pain** | 2/28 (7.1%) [Kobyliak2018]  1/25 (4.0%) [Ahmed2018]  1/16 (6.3%) [Chong2021] |  | 1/85 (1.2%) [Won2026] | NA | 1/26 (3.8%) [Eslamparast2014] | 2/25 (8.0%) [Ahmed2018] | NA | NA | Mild |
| **Abdominal cramps** | 1/16 (6.3%) [Chong2021] |  | NA | NA | NA | NA | NA | NA | Mild |
| **Constipation** | 1/25 (4.0%) [Ahmed2018] |  | 6/85 (7.1%) [Won 2026] | NA | NA | 1/25 (4.0%) [Ahmed2018] | NA | NA | Mild |
| **Dyspepsia** | 1/10 (10.0%) [Wong2013] |  | 2/10 (20.0%) [Wong2013] | NA | NA | NA | NA | NA | Mild |
| **Aversion to taste/smell** | NA |  | 2/24 (8.3%) [Crommen2022] | NA | NA | NA | NA | NA | Mild |
| **Rash** | 1/10 (10.0%) [Wong2013] |  | 1/19 (5.3%) [Chong2021]  1/85 (1.2%) [Won 2026] | NA | NA | NA | NA | NA | Mild |
| **Headache** | NA |  | 1/30 (3.3%) [Kobyliak2018] | NA | 1/26 (3.8%) [Eslamparast2014] | 1/25 (4.0%) [Ahmed2018] | NA | NA | Mild to moderate |
| **Dizziness** | 1/25 (4.0%) [Ahmed2018] |  | NA | NA | NA | 1/25 (4.0%) [Ahmed2018] | NA | NA | Mild |
| **Back/neck pain** | 1/16 (6.3%) [Chong2021]  1/25 (4.0%) [Ahmed2018] |  | 2/25 (8.0%)  [Ahmed2018] | NA | NA | 2/25 (8.0%)  [Ahmed2018] | NA | NA | Mild |
| **Musculoskeletal pain** | 1/10 (10.0%) [Wong2013] |  | 2/10 (20.0%)  [Wong2013] | NA | NA | NA | NA | NA | Mild |
| **Chest pain** | NA |  | NA | NA | NA | 2/25 (8.0%) [Ahmed2018] | NA | NA | Mild |
| **Urinary tract infection** | NA |  | 3/19 (15.8%)  [Chong2021] | NA | NA | NA | NA | NA | Mild to moderate |
| **Genital thrush** | NA |  | 1/19 (5.3%)  [Chong2021] | NA | NA | NA | NA | NA | Mild to moderate |
| **Traumatic toe infection** | 1/16 (6.3%) [Chong2021] |  | NA | NA | NA | NA | NA | NA | Mild to moderate |
| **Cold** | Reported (no specific n) [Crommen2022] |  | Reported (no specific n) [Crommen2022] | NA | NA | NA | NA | NA | Mild |
| **Pneumonia** | 1/25 (4.0%) [Ahmed2018] |  | NA | NA | NA | NA | NA | NA | Serious adverse event |
| **Cough** | 1/10 (10.0%) [Wong2013] |  | NA | NA | NA | NA | NA | NA | Mild |
| **Mastalgia** | NA |  | NA | NA | NA | 2/25 (8.0%) [Ahmed2018] | NA | NA | Mild |
| **Hematochezia** | NA |  | NA | NA | NA | 1/25 (4.0%) [Ahmed2018] | NA | NA | Mild |
| **Peripheral edema** | 2/25 (8.0%) [Ahmed2018] |  | NA | NA | NA | NA | NA | NA | Mild |
| **Duodenal ulcer** | NA |  | NA | NA | NA | 1/25 (4.0%) [Ahmed2018] | NA | NA | Serious adverse event |
| **Overall Incidence** | **19/179 (10.6%)** |  | **29/229 (12.7%)** | **0/29 (0%)** | **1/26 (3.8%)** | **14/25 (56.0%)** | **0/50 (0%)** | **0/34 (0%)** | **Mostly mild** |

NA indicates one of two scenarios for that specific adverse event: (a) the event was not reported in studies that otherwise provided safety data for that intervention group, or (b) the original study explicitly reported no occurrence of this event.

Total Incidence Calculation: The numerator is the sum of unique patients experiencing any adverse event within each group across all studies that provided safety data (including those reporting zero events). The denominator is the sum of total participants for that group in those same studies. Studies that did not report any safety assessment data were excluded from this calculation. This provides a conservative estimate based on available reports.

Supplementary Tabel 7 Summary of comprehensive risk of bias

| **Author /year** | **Random sequence generation**  **(selection bias)** | **Allocation concealment (selection bias)** | **Blinding of participants and personnel (performance bias)** | **Blinding of outcome assessment**  **(detection bias)** | **Incomplete outcome data (attrition bias)** | **Selective reporting (reporting bias)** | **Other bias** |
| --- | --- | --- | --- | --- | --- | --- | --- |
| Abhari2020 | Low risk risk | Low risk | Low risk | Low risk | Low risk | Low risk | Low risk |
| Ahmed2018 | Low risk | Low risk | Low risk | Low risk | Low risk | Low risk | Low risk |
| Alisi2014 | Low risk | Low risk | Low risk | Low risk | Low risk | Low risk | Low risk |
| Behrouz2020 | Low risk | Low risk | Low risk | Low risk | Low risk | Low risk | Low risk |
| Chong2020 | Low risk | Low risk | Low risk | Low risk | Low risk | Low risk | Low risk |
| Chong2021 | Low risk | Low risk | Low risk | Low risk | Low risk | Low risk | Low risk |
| Crommen2022 | Low risk | Low risk | Low risk | Low risk | Low risk | Low risk | Low risk |
| Didyk2024 | Low risk | Unclear risk (The methodology was not explicitly described in the original article, leading to an “Unclear risk” assessment for Allocation concealment (selection bias).) | Unclear risk (The methodology was not explicitly described in the original article, leading to an “Unclear risk” assessment for Blinding of participants and personnel (performance bias).) | Low risk | Low risk | Low risk | Low risk |
| Escouto2023 | Low risk | Low risk | Low risk | Low risk | Low risk | Low risk | Low risk |
| Eslamparast2014 | Low risk | Low risk | Low risk | Low risk | Low risk | Low risk | Low risk |
| Famouri2017 | Low risk | Low risk | Low risk | Low risk | Low risk | Low risk | Low risk |
| Ferolla2016 | Low risk | Low risk | Low risk | Low risk | Low risk | Low risk | Low risk |
| Fogacci2024 | Low risk | Low risk | Low risk | Low risk | Low risk | Low risk | Low risk |
| Hamid2024 | Low risk | Low risk | Unclear risk (The methodology was not explicitly described in the original article, leading to an “Unclear risk” assessment for Blinding of participants and personnel (performance bias).) | Low risk | Low risk | Low risk | Low risk |
| Kobyliak2018 | Low risk | Low risk | Low risk | Low risk | Low risk | Low risk | Low risk |
| Manzhalii2017 | Low risk | Low risk | High risk (The study was explicitly described as a “non-blinded” trial.) | Low risk | Low risk | Low risk | Low risk |
| Mofidi2017 | Low risk | Low risk | Low risk | Low risk | Low risk | Low risk | Low risk |
| Mohamad2021 | Low risk | Low risk | Low risk | Low risk | Low risk | Low risk | Low risk |
| Mohammadi2025 | Low risk | Low risk | High risk (Although the study mentions adherence to CONSORT, the authors stated a “lack of blindness” as a study limitation.) | Low risk | Low risk | Low risk | Low risk |
| Nabavi2014 | Low risk | Low risk | Low risk | Low risk | Low risk | Low risk | Low risk |
| Reshef2024 | Low risk | Low risk | Low risk | Low risk | Low risk | Low risk | Low risk |
| Savytska2025 | Low risk | Low risk | Low risk | Low risk | Low risk | Low risk | Low risk |
| Sayari2018 | Low risk | Low risk | Low risk | Low risk | Low risk | Low risk | Low risk |
| Sepideh2015 | Low risk | Low risk | Low risk | Low risk | Low risk | Low risk | Low risk |
| Won2026 | Low risk | Low risk | Low risk | Low risk | Low risk | Low risk | Low risk |
| Wong2013 | Low risk | Low risk | Low risk | Low risk | Low risk | Low risk | Low risk |
| Yazdani2025 | Low risk | Low risk | Low risk | Low risk | Low risk | Low risk | Low risk |

Supplementary 8 Full list of MDs and 95%CIs for all pairwise comparisons across all outcomes

| **Treatment Comparison** | **ALT** | **AST** | **GGT** | **CAP** | **LDL-C** | **HDL-C** | **TC** | **TG** | **TNF-ɑ** | **IL-6** |
| --- | --- | --- | --- | --- | --- | --- | --- | --- | --- | --- |
| **vs A (Placebo)** |  |  |  |  |  |  |  |  |  |  |
| **B vs A** | -7.51 (-12.36, -2.66) | -6.42 (-11.91, -0.92) | -2.08 (-11.36, 7.20) | -10.98 (-39.09, 17.13) | -0.11 (-0.38, 0.16) | 0.00 (-0.18, 0.18) | -0.47 (-0.99, 0.05) | -0.13 (-0.28, 0.03) | -1.05 (-4.08, 6.17) | -0.73 (-2.96, 4.43) |
| **C vs A** | -13.64 (-27.07, -0.22) | -3.73 (-17.29, 9.82) | -12.49 (-31.59, 6.62) | NA | -0.50 (-1.10, 0.09) | -0.13 (-0.53, 0.28) | -0.55 (-1.78, 0.67) | -0.24 (-0.64, 0.17) | NA | NA |
| **D vs A** | -6.24 (-12.68, 0.21) | -13.13 (-20.82, -5.45) | -12.40 (-23.13, -1.68) | -45.69 (-56.39, -34.99) | -0.40 (-0.76, -0.03) | 0.16 (-0.06, 0.38) | -0.37 (-1.17, 0.44) | 0.15 (-0.09, 0.38) | -1.60 (-3.19, 6.39) | NA |
| **E vs A** | -24.30 (-47.02, -1.58) | -13.80 (-35.88, 8.28) | -18.80 (-44.49, 6.89) | NA | -0.43 (-1.26, 0.39) | 0.00 (-0.55, 0.55) | -0.22 (-2.02, 1.59) | 0.04 (-0.52, 0.59) | -5.51 (-14.95, 3.93) | -1.31 (-6.84, 4.22) |
| **F vs A** | -15.40 (-35.04, 4.24) | -7.46 (-26.91, 11.99) | -16.63 (-46.14, 12.88) | NA | 0.43 (-0.44, 1.30) | 0.18 (-0.44, 0.80) | -0.42 (-2.22, 1.39) | -0.13 (-0.68, 0.43) | NA | NA |
| **G vs A** | -7.75 (-19.86, 4.36) | -9.55 (-23.06, 3.96) | NA | 3.70 (-30.58, 37.98) | NA | NA | 0.20 (-1.62, 2.03) | -0.08 (-0.70, 0.54) | NA | -4.20 (-9.68, 1.28) |
| **Active Comparisons** |  |  |  |  |  |  |  |  |  |  |
| **C vs B** | -6.13 (-19.59, 7.33) | 2.68 (-11.27, 16.64) | -10.41 (-30.09, 9.27) | NA | -0.39 (-1.00, 0.22) | -0.13 (-0.54, 0.28) | -0.08 (-1.35, 1.18) | -0.11 (-0.52, 0.30) | NA | NA |
| **D vs B** | 1.27 (-6.80, 9.35) | -6.72 (-16.16, 2.73) | -10.33 (-24.51, 3.86) | -34.71 (-64.79, -4.64) | -0.29 (-0.74, 0.17) | 0.16 (-0.13, 0.44) | 0.10 (-0.86, 1.06) | 0.27 (-0.01, 0.56) | -0.55 (-6.46, 7.56) | NA |
| **E vs B** | -16.79 (-40.02, 6.44) | -7.38 (-30.13, 15.37) | -16.72 (-44.03, 10.59) | NA | -0.33 (-1.19, 0.54) | 0.00 (-0.59, 0.58) | 0.25 (-1.62, 2.13) | 0.16 (-0.41, 0.74) | -6.56 (-17.30, 4.19) | -2.04 (-8.70, 4.61) |
| **F vs B** | -7.89 (-28.12, 12.34) | -1.04 (-21.25, 19.16) | -14.55 (-45.49, 16.39) | NA | 0.54 (-0.37, 1.45) | 0.18 (-0.47, 0.83) | 0.06 (-1.82, 1.93) | 0.00 (-0.58, 0.57) | NA | NA |
| **G vs B** | -0.24 (-13.36, 12.88) | -3.13 (-17.71, 11.44) | NA | 14.68 (-29.65, 59.01) | NA | NA | 0.67 (-1.22, 2.57) | 0.05 (-0.59, 0.69) | NA | -4.93 (-11.54, 1.68) |
| **D vs C** | 7.41 (-7.49, 22.31) | -9.40 (-24.98, 6.18) | 0.08 (-21.81, 21.97) | NA | 0.10 (-0.59, 0.80) | 0.29 (-0.17, 0.75) | 0.19 (-1.28, 1.65) | 0.38 (-0.09, 0.85) | NA | NA |
| **E vs C** | -10.66 (-37.05, 15.73) | -10.07 (-35.97, 15.84) | -6.31 (-38.33, 25.70) | NA | 0.07 (-0.95, 1.08) | 0.13 (-0.56, 0.81) | 0.34 (-1.84, 2.52) | 0.27 (-0.41, 0.96) | NA | NA |
| **F vs C** | -1.76 (-25.54, 22.03) | -3.73 (-27.43, 19.98) | -4.14 (-39.30, 31.01) | NA | 0.93 (-0.12, 1.99) | 0.31 (-0.43, 1.05) | 0.14 (-2.04, 2.32) | 0.11 (-0.58, 0.80) | NA | NA |
| **G vs C** | 5.89 (-12.30, 24.09) | -5.81 (-24.96, 13.34) | NA | NA | NA | NA | 0.76 (-1.44, 2.95) | 0.16 (-0.58, 0.90) | NA | NA |
| **E vs D** | -18.06 (-41.68, 5.55) | -0.67 (-24.04, 22.71) | -6.40 (-34.23, 21.44) | NA | -0.04 (-0.94, 0.86) | -0.16 (-0.76, 0.43) | 0.15 (-1.82, 2.12) | -0.11 (-0.71, 0.49) | -7.11 (-17.70, 3.48) | NA |
| **F vs D** | -9.16 (-29.83, 11.51) | 5.67 (-15.24, 26.58) | -4.23 (-35.63, 27.18) | NA | 0.83 (-0.12, 1.77) | 0.02 (-0.64, 0.68) | -0.05 (-2.02, 1.93) | -0.27 (-0.88, 0.33) | NA | NA |
| **G vs D** | -1.51 (-15.22, 12.19) | 3.59 (-11.96, 19.13) | NA | 49.39 (13.48, 85.30) | NA | NA | 0.57 (-1.42, 2.56) | -0.22 (-0.89, 0.44) | NA | NA |
| **F vs E** | 8.90 (-21.13, 38.93) | 6.34 (-23.08, 35.76) | -2.17 (-36.96, 41.30) | NA | 0.86 (-0.34, 2.06) | 0.18 (-0.65, 1.02) | -0.20 (-2.75, 2.35) | -0.17 (-0.95, 0.62) | NA | NA |
| **G vs E** | 16.55 (-9.20, 42.30) | 4.25 (-21.63, 30.13) | NA | NA | NA | NA | 0.42 (-2.15, 2.98) | -0.11 (-0.95, 0.72) | NA | -2.89 (-10.68, 4.90) |
| **G vs F** | 7.65 (-15.42, 30.72) | -2.09 (-25.77, 21.59) | NA | NA | NA | NA | 0.62 (-1.95, 3.18) | 0.05 (-0.78, 0.88) | NA | NA |

Intervention abbreviations: A= placebo or usual care controls; B= probiotics; C= prebiotics; D= synbiotics; E= antibiotics; F= postbiotics; G= combination of antibiotics and gut microbiota-regulating drugs (combination treatment).

Supplementary Tabel 9 Meta-regression analysis

| **Covariates** | **Endpoint** | **Comparison** | **Beta coefficients** | **95%CI** |
| --- | --- | --- | --- | --- |
| Intervention duration | ALT | A vs B | 3.254 | [-13.182]-[20.3013] |
|  |  | A vs C | -13.406 | [-285.797]-[153.9505] |
|  |  | A vs D | -6.731 | [-19.811]-[6.2925] |
|  |  | A vs E | 2.579 | [ -43.358]-[58.7853] |
|  |  | A vs F | 10.548 | [-459.743]-[571.7893] |
|  |  | A vs G | 3.9 | [-253.212]-[312.5367] |
|  | AST | A vs B | -3.8236 | [-19.301]-[10.7876] |
|  |  | A vs C | -9.3117 | [-328.690]-[260.1696] |
|  |  | A vs D | -7.3276 | [-25.131]-[9.6737] |
|  |  | A vs E | 45.6201 | [-198.234]-[845.5642] |
|  |  | A vs F | -0.4606 | [-186.679]-[177.5980] |
|  |  | A vs G | 13.0125 | [-14.333]-[40.7844] |
|  | GGT | A vs B | -0.555 | [-29.432]-[29.253] |
|  |  | A vs C | 60.58 | [-261.423]-[1785.711] |
|  |  | A vs D | -8.156 | [-30.580]-[14.755] |
|  |  | A vs E | -30.416 | [-464.771]-[194.574] |
|  |  | A vs G | 23.89 | [-246.816]-[514.734] |
|  | CAP | A vs B | -8.8965 | [-374.7873]-[293.752] |
|  |  | A vs D | -0.1819 | [-56.3044]-[55.728] |
|  |  | A vs G | -40.8247 | [-1020.8582]-[329.682] |
|  | LDL-C | A vs B | 1.733 | [-8.0307]-[11.256] |
|  |  | A vs C | -4.825 | [-264.2033]-[224.149] |
|  |  | A vs D | -4.433 | [-23.9957]-[14.954] |
|  |  | A vs F | 14.404 | [-390.8358]-[557.340] |
|  |  | A vs G | 3.386 | [-141.0996]-[168.499] |

Intervention abbreviations: A= placebo or usual care controls; B= probiotics; C= prebiotics; D= synbiotics; E= antibiotics; F= postbiotics; G= combination of antibiotics and gut microbiota-regulating drugs (combination treatment).

Supplementary Figure 1 Forest plot of TC


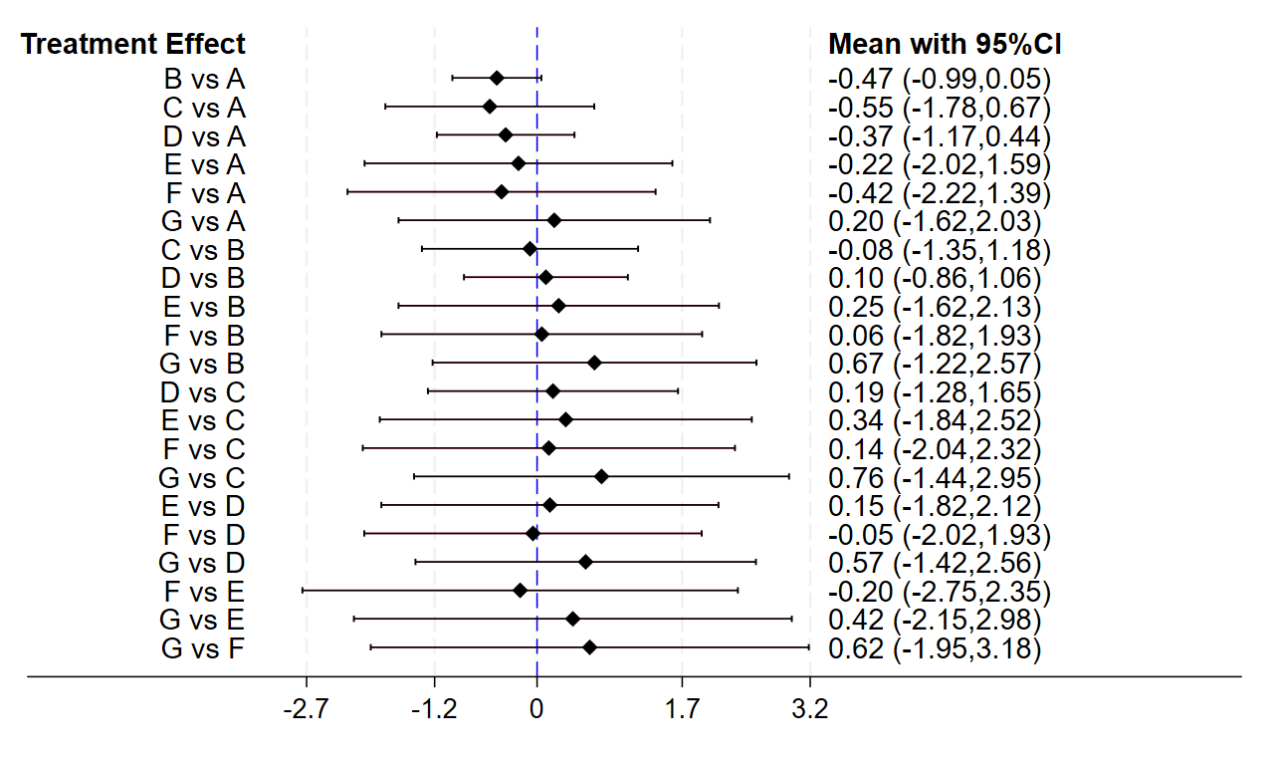


Supplementary Figure 2 TC SUCRA ranking


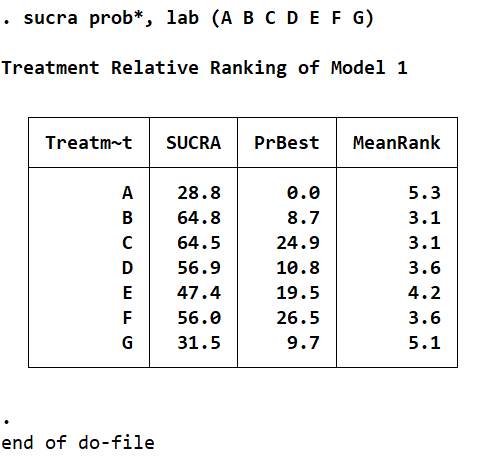


Supplementary Figure 3 Forest plot of TG


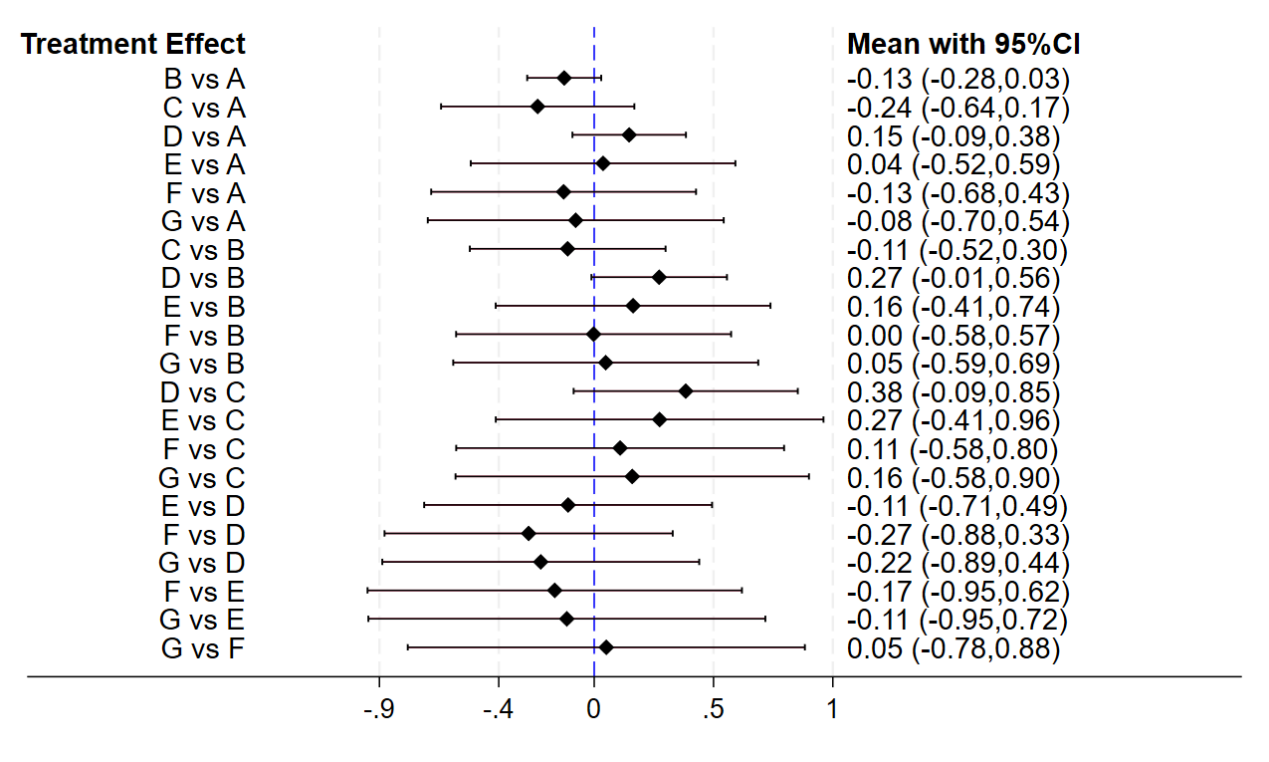


Supplementary Figure 4 TG SUCRA ranking


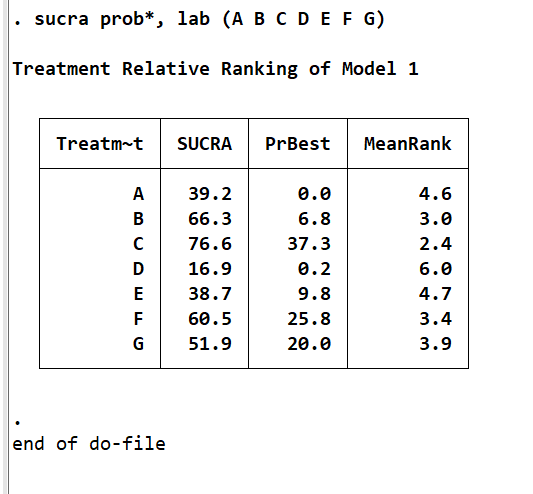


Supplementary Figure 5 Forest plot of HDL-C


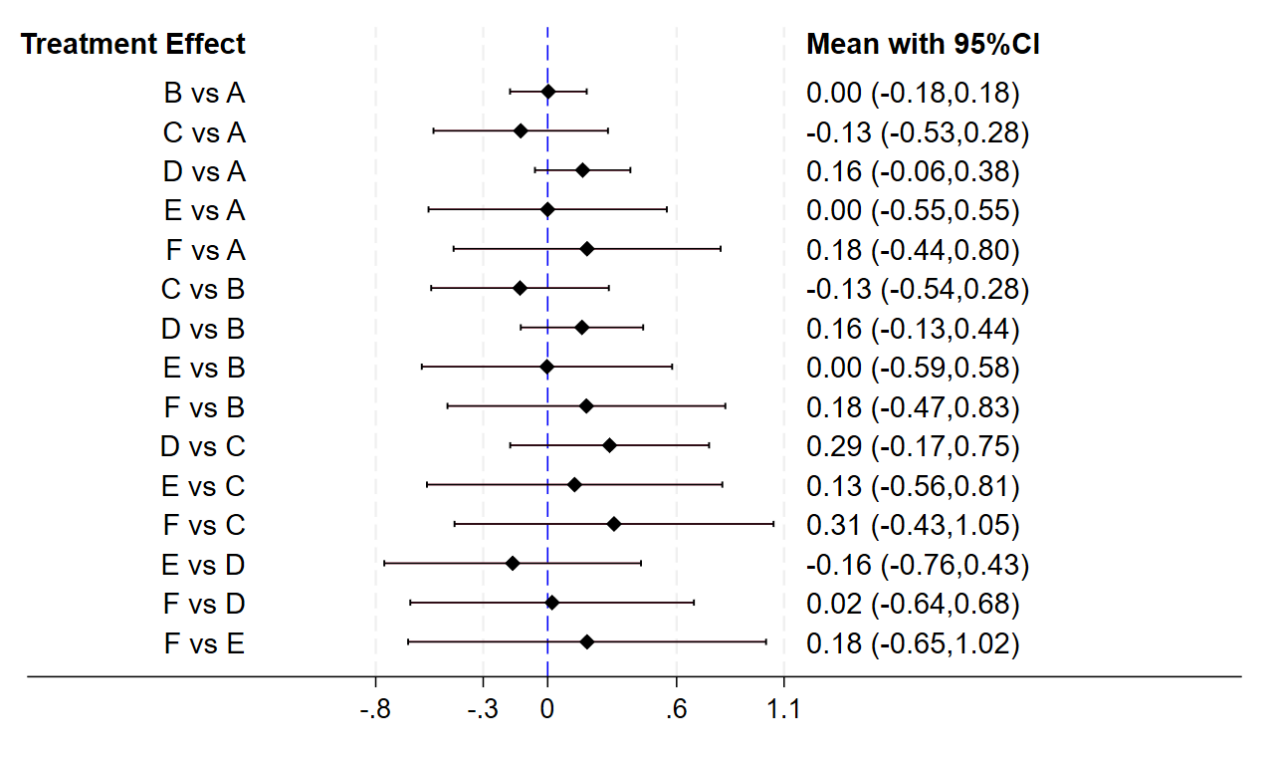


Supplementary Figure 6 HDL-C SUCRA ranking


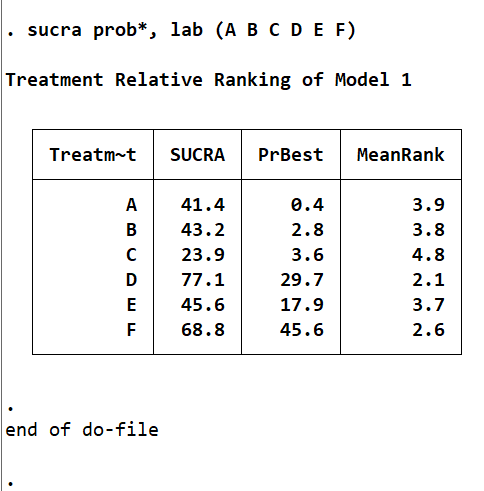


Supplementary Figure 7 Forest plot of TNF-α


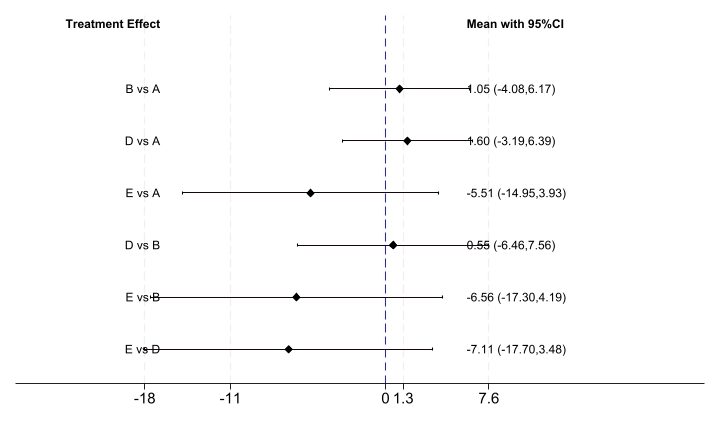


Supplementary Figure 8 TNF-α SUCRA ranking


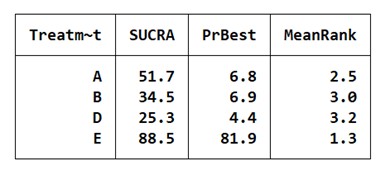


Supplementary Figure 9 Forest plot of IL-6


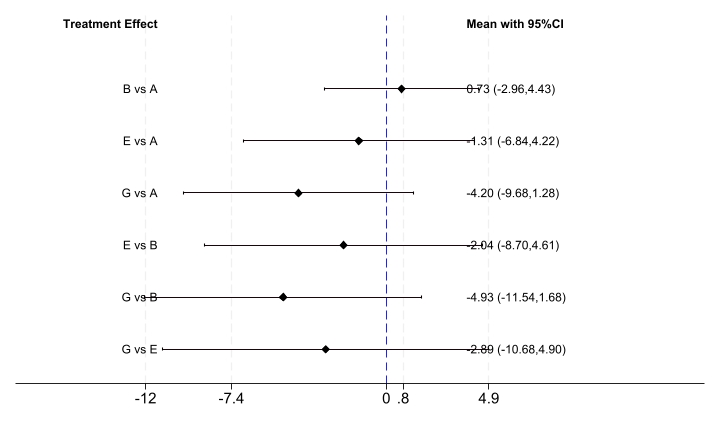


Supplementary Figure 10 IL-6 SUCRA ranking


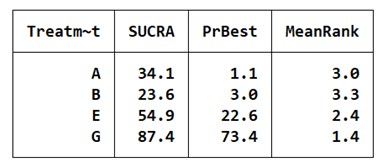


Supplementary Text

Search formula for each database:

Pubmed: (probiotics OR prebiotics OR synbiotics OR postbiotics OR antibiotics) and ("non-alcoholic fatty liver disease" OR “NAFLD” OR "metabolic associated fatty liver disease" OR “MAFLD” OR "metabolic dysfunction-associated steatotic liver disease" OR “MASLD”)

Web of Science: (probiotics OR prebiotics OR synbiotics OR postbiotics OR antibiotics) and ("non-alcoholic fatty liver disease" OR “NAFLD” OR "metabolic associated fatty liver disease" OR “MAFLD” OR "metabolic dysfunction-associated steatotic liver disease" OR “MASLD”)

Embase: ('probiotics'/exp OR probiotics OR 'prebiotics'/exp OR prebiotics OR 'synbiotics'/exp OR synbiotics OR 'postbiotics'/exp OR postbiotics OR 'antibiotics'/exp OR antibiotics) AND ('non-alcoholic fatty liver disease'/exp OR 'non-alcoholic fatty liver disease' OR 'nafld' OR 'metabolic associated fatty liver disease'/exp OR 'metabolic associated fatty liver disease' OR 'mafld' OR 'metabolic dysfunction-associated steatotic liver disease' OR 'masld')

Cochrane: (probiotics OR prebiotics OR synbiotics OR postbiotics OR antibiotics) and ("non-alcoholic fatty liver disease" OR “NAFLD” OR "metabolic associated fatty liver disease" OR “MAFLD” OR "metabolic dysfunction-associated steatotic liver disease" OR “MASLD”)

All papers have been issued in English-language journal.

Supplementary raw Stata code

1.network setup mean sd n, study(id) trt(t)format(augment)

network setup mean sd n, study(id) trt(t)format(augment) smd

2.network map

3.network meta i

4.network meta c, force

network sidesplit all, tau

5.network rank min, all zero reps(5000) gen(prob)

6.sucra prob*, lab (A B C D E G)

7.netleague, lab (A B C D E G) sort (E C G D B A) export ("D:\network.xlsx")

8.intervalplot, null(0) lab(A B C D E G) textsize(medium)

9.network convert pairs

netfunnel _y _stderr _t1 _t2, random bycomp add(lfit _stderr _ES_CEN) noalpha

10.ifplot _y _stderr _t1 _t2 id, tau2(loop)

11.netweight _y _stderr _t1 _t2
